# Position-specific propensities of amino acids in the *β*-strand

**DOI:** 10.1186/1472-6807-10-29

**Published:** 2010-09-28

**Authors:** Nicholus Bhattacharjee, Parbati Biswas

**Affiliations:** 1 Department of Chemistry, University of Delhi, Delhi - 110007, India

## Abstract

**Background:**

Despite the importance of *β*-strands as main building blocks in proteins, the propensity of amino acid in *β*-strands is not well-understood as it has been more difficult to determine experimentally compared to *α*-helices. Recent studies have shown that most of the amino acids have significantly high or low propensity towards both ends of *β*-strands. However, a comprehensive analysis of the sequence dependent amino acid propensities at positions between the ends of the *β*-strand has not been investigated.

**Results:**

The propensities of the amino acids calculated from a large non-redundant database of proteins are found to be highly position-specific and vary continuously throughout the length of the *β*-strand. They follow an unexpected characteristic periodic pattern in inner positions with respect to the cap residues in both termini of *β*-strands; this periodic nature is markedly different from that of the *α*-helices with respect to the strength and pattern in periodicity. This periodicity is not only different for different amino acids but it also varies considerably for the amino acids belonging to the same physico-chemical group. Average hydrophobicity is also found to be periodic with respect to the positions from both termini of *β*-strands.

**Conclusions:**

The results contradict the earlier perception of isotropic nature of amino acid propensities in the middle region of *β*-strands. These position-specific propensities should be of immense help in understanding the factors responsible for *β*-strand design and efficient prediction of *β*-strand structure in unknown proteins.

## Background

Secondary structural elements like *α*-helices and *β*-strands are important determinants of folded protein structure and topology. Helices and strands are regular repetitive structures; while *α*-helices are quasi-one-dimensional formed by local interactions [1,2], long *β*-strands self-assemble into complex hydrogen-bonded *β*-sheets by long-range and inter-chain interactions [3-5]. Secondary structures are predicted on the basis of statistical analysis of known protein structures, fold recognition and multiple sequence alignments. Various close packing arrangements of these strands and helices are systematically optimized [6] to test the resultant tertiary structure or a specific fold. It is, therefore, important to understand the factors dictating the intrinsic preferences of amino acid residues for a particular secondary structure [7].

Statistical analysis of known proteins [7,8] clearly reveals that amino acids have definite conformational preferences for one or the other type of secondary structure. Secondary structure prediction methods [9-13] systematically analyze how these preferences determine whether a given sequence will adopt an *α*-helical or a *β*-sheet topology or neither. Even the frequencies of occurrences of amino acid residues in a helix at the N-terminus end (N-cap), at the C-terminus end (C-cap) and at interior positions are very different [14-25]. This non-equivalence of different positions around the helix termini with respect to amino acid preferences is also supported by experimental results [26-36]. Though early studies establish distinct differences in the propensities of the amino acids at N-cap, N1, N2 and N3 positions [14,15,37-39], it was assumed that beyond the first few residues from both the termini, the individual propensities average out leading to essentially isotropic environments [16]. An unexpected recent finding confirms that the sequence dependence of helical propensities at positions between the ends of helices are markedly different and they exhibit a distinct pattern throughout the helix length [40].

Despite the importance of *β*-strands as main building blocks in proteins, the propensity of *β*-strands is not well-understood as it has been more difficult to determine experimentally compared to *α*-helices. This is attributed to the fact that *β*-sheets do not fold independently. Another reason may be the structural context dependence of the amino acids in *β*-sheet formation. A statistical survey of the protein structure database correlates well with an average of the experimental scales to determine the *β*-sheet propensity [41] and supports the idea that the intrinsic *β*-sheet propensity plays a pivotal role in assessing protein stability [42]. Various factors like the side-chain dependent steric interactions [43] and solvent screening of the backbone electrostatic interactions [44] dictate the preference of the amino acids for *β*-sheet formation. Conformational entropy analysis also quantitatively establishes [45] the role of steric clashes between the side-chain and local backbone of an amino acid as the dominant cause of intrinsic *β*-sheet propensity. Recently, it has been proved that even the conformational topology of the backbone influences the propensity of amino acids in *β*-sheets [46,47]. These extensive analyses, however, do not reveal a clear and concise rationale of *β*-sheet propensity distribution and are far from being fully conclusive.

A recent study demonstrates that the different positions in the *β*-sheet are not isotropic with respect to amino acid propensities. There is a marked variation in the pattern of amino acid preferences in different positions around the N-cap and C-cap region of the *β*-sheet [48]. However, a comprehensive analysis of the sequence dependent *β*-strand propensities at positions between the ends of the *β*-strand has not been investigated. In this article, we present a detailed and systematic position-wise dissection of the amino acid propensities in the different subregions of *β*-strands. We note that the inner positions of the *β*-strand exhibit an unexpected characteristic periodicity in the sequence-dependent propensities which is distinctly different from that of *α*-helices with respect to both strength and pattern in periodicity. Average hydrophobicity also follows a similar position dependent periodic pattern throughout the different regions of the *β*-strand. This work may have far-reaching implications on the formation and stability of *β*-strands and provide the necessary foundation both for improvising new secondary structure prediction algorithms and *de novo *protein design.

## Methods

### Database

A non-redundant database of *β*-strand sequences was compiled from the May-2008 release of PDB-select [49]. All protein chains in this database have sequence identity of ≤ 25%. High resolution protein structures determined by X-ray crystallography with resolution higher than 3Å and R-factor ≤ 0.3 were selected. The database consisted of a total of 2586 non-redundant protein chains from 2466 proteins. The secondary structure assignment of these protein chains was performed with the help of DSSP [50], which is the most widely used methodology to define secondary structures of proteins from experimentally determined tertiary structures. DSSP classifies the amino acids of a particular protein chain into 8 types of secondary structure classes: H(*α*-helix), G(3_10_-helix), I(*π*-helix), E(*β*-strand), B(isolated *β*-bridge), T(turn), S(bend) and -(rest). In this work, E and B were annotated with *β*-strand conformation. The final database consisted of 15579 *β*-strands. Isolated *β*-bridges (annotated as B in DSSP) with length greater than 3 residues were not found in the database. In this work, *β*-strands were not differentiated as constituent strands of *β*-sheet or isolated *β*-bridges. DSSP predicts the residue numbers of the complementary strands constituting the hydrogen bonded *β*-strands which form the *β*-sheet structure. Each *β*-strand was designated to be part of parallel or antiparallel *β*-sheets if both the complementary strands are either parallel or antiparallel respectively. If one strand is parallel and the complementary strand is antiparallel then the strand was designated to be part of a mixed *β*-sheet structure [51]. To verify that the results of this study are independent of any database bias, an additional database of *β*-strands from *β*-barrel proteins were also compiled (with sequence identity ≤ 25%, resolution ≤ 3Å and crystallographic R-factor ≤ 0.3).

DSSP considers H-bonds for the assignment of helices and sheets. In case of helices, N-Cap is referred to the first residue preceding the helix which is in non-helical conformation while C-Cap is assigned as the first residue succeeding the helix which is in non-helical conformation [15,40]. Similarly N-Cap is the residue preceding the first *β*-strand residue i.e. N1 while C-Cap is the residue succeeding the last residue in *β*-strand i.e. C1 [37,52]. According to this analysis, N-Cap and C-Cap residues were numbered as zero in the figures while the inner residues in the assigned *β*-strand range from N1 to N10 and C1 to C10.

### Propensity

Propensity, which is also referred to as conformational parameter, is used to quantify the intrinsic preference of a given amino acid for a specific position in a particular secondary structure [7]. A position-wise analysis of the amino acid propensity from both N and C termini of the *β*-strand is performed. Position-specific propensity of an amino acid is defined as [16,40]

(1) $$P_{ij}=\frac{f_{ij}}{f_{i}}=\frac{n_{ij}/\sum n_{ij}}{N_{i}/\sum N_{i}}$$

where *n_ij _*and *f_ij _*are the number and fraction of the *i^th ^*residue in *j^th ^*position while *n_i _*and *f_i _*are the number and fraction of finding *i^th ^*residue in whole database of 2586 non-redundant protein chains respectively. The summation is over *i *for the 20 amino acids. From this equation, it can be clearly seen that a value of propensity greater than one indicates a higher preference of the amino acid in that position whereas a value less than one implies lower preference of that amino acid [41].

### *χ*^2^**-values**

The *χ*^2 ^values at *j^th ^*positions of *β*-strands are defined as

(2) $$\chi_{j}^{2}=\sum_{i=1}^{20}\frac{{\left(n_{ij}-nexpec_{ij}\right)}^{2}}{nexpec_{ij}}$$

where *n_ij _*is the observed number of *i^th ^*amino acid at *j^th ^*position while *nexpec_ij _*is the expected number of *i^th ^*amino acid at *j^th ^*position. The expected number of amino acid, *nexpec_ij _*, at a given position *j *in strand is evaluated as

(3) $$nexpec_{ij}=\frac{r_{i}N_{j}}{R}$$

where *r_i _*is the number of *i^th ^*amino acid in the reference distribution, *N_j _*number of amino acids at the *j^th ^*position of the *β*-strand and *R *is the total number of amino acids in the reference distribution (distribution of amino acids in *β*-strands).

### Hydrophobicity

Local hydrophobicity is found to play a dominant role in the stabilization of secondary structures in proteins [53]. Different scales are found to predict significantly different hydrophobicities with residues being strongly hydrophobic on one scale and mildly hydrophobic on another. In most cases, hydrophobicity is measured by the free energy change due to the transfer of a non polar solute into aqueous solution at a particular characteristic temperature [54,55]. The average hydrophobicity of *j^th ^*position in *β*-strand, *Hb_j _*, is calculated as [40]

(4) $$Hb_{j}=\sum_{i=1}^{20}\Delta G_{i}^{cor}\frac{n_{ij}}{n_{j}}$$

where *n_ij _*and *n_j _*are the number of *i^th ^*residues at *j^th ^*position and the number of total residues at *j^th ^*position respectively. $\Delta G_{i}^{cor}$ is the experimentally measured free energy change resulting from the transfer of the *i^th ^*amino acid from octanol to water [56].

### Free energy

Most of the experimental propensity scales are based on free energy differences. The propensities of amino acids may be converted into a free energy like term by the following equation [41]

(5) $$E\left(i,\;j\right)=-log\left[P_{ij}\right]$$

where *E*(*i, j*) is the free energy of the *i^th ^*amino acid residue at the *j^th ^*position and *P_ij _*is the propensity calculated by eqn.(1). A very similar free energy criteria was used earlier to study the pairing of residues in the neighbouring strands of *β*-sheets [57] as well as ranking of various factors which contribute to *β*-sheet folding [58].

## Results and Discussion

### Number distribution of *β*-strands

**Figure 1:**
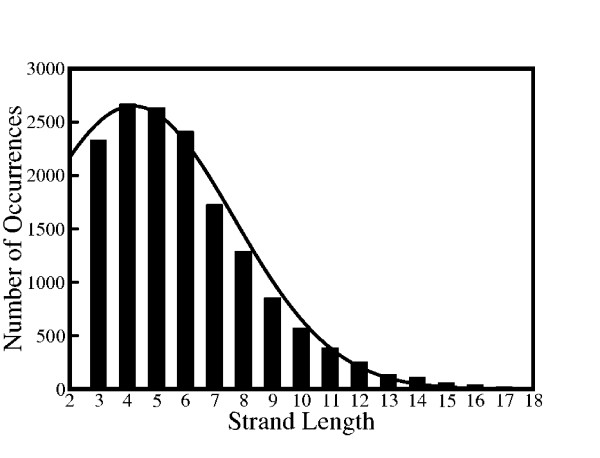
**Occurrence Frequency of ***β***-strands**. Frequency of occurrence of a given strand length in the non-redundant protein database. A Gaussian curve is fitted to the plot. This fit has a center at 4.2 residues, a width of 4.9 residues and an amplitude of 2650 occurrences.

Figure 1 illustrates the frequency of occurrence of strands as a function of the strand length in the chosen database. In agreement with earlier studies, the peak is observed near the strand length N = 5 residues [59]. The number of occurrences fall off very sharply above the strand length of 15 residues which constitutes less than 1% of total strand population. Unlike helices, the number of occurrence of *β*-strands with respect to a specific strand length shows a minimal deviation from the fitted Gaussian curve.

### Propensities of amino acids show significant deviation from the isotropic nature at middle region of *β*-strands

**Figure 2:**
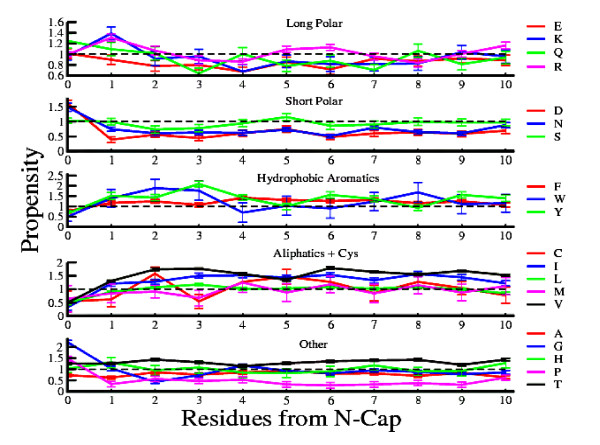
**Position-Specific Propensities from N-terminus**. Position-specific propensities for each amino acid in the first 10 strand positions from the N-terminus. Position-wise propensities for each *β*-strands are calculated and standard errors of the data are plotted as error bars.

**Figure 3:**
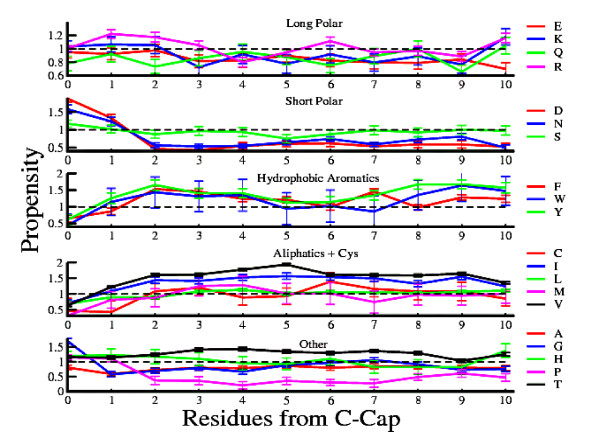
**Position-Specific Propensities from C-terminus**. Position-specific propensities for each amino acid in the first 10 strand positions from the C-terminus. Position-wise propensities for each *β*-strands are calculated and standard errors of the data are plotted as error bars.

Propensities of 20 amino acids are examined for 10 inner positions from the cap residues in *β*-strand. Strands of length 10 residues or more are used for this study. A higher cut-off length is not possible as the number of strands significantly decreases beyond 15 residues as mentioned above. There are 1634 such strands in the chosen database. The position-specific physico-chemical properties are calculated considering this data-set of strands. The position-specific propensities of the amino acids in these *β*-strands are calculated according to equation 1. Figures 2 and 3 illustrate these position-specific propensities of amino acids from both termini of *β*-strands. The Figures are generated by dividing 20 amino acids into five groups namely (i)long polar (E, K, Q, R), (ii)short polar (D, N, S), (iii)hydrophobic aromatics (F, W, Y), (iv)aliphatics + cysteine (C, I, L, M, V) and (v)other (A, G, H, P, T) in accordance to their physico-chemical properties [40]. The error bars in the figures depict the standard errors obtained by calculating the position-specific propensities of the amino acids in individual *β*-strands.

A recent study demonstrates that the propensities of amino acids are independent of positions in the middle region of *β*-strands [48]. The results from this study exhibit a striking contrast to such an expected isotropic pattern of propensities. Figures 2 and 3 show a significant variation of the propensity in the inner positions of the *β*-strands. Even the neighbouring positions are found to possess appreciable differences in their propensity values for a given amino acid. For example, methionine at N3 position has propensity 0.67 ± 0.3 and at N4 its propensity is 1.25 ± 0.3. The corresponding numbers for glutamine are 0.64 ± 0.09 and 1.00 ± 0.1 respectively and that of tryptophan are 1.76 ± 0.4 and 0.70 ± 0.4. Another significant feature of Figures 2 and 3 is the periodicity. Propensities of the amino acids display a characteristic periodic behaviour with respect to positions from the cap residues in *β*-strands. However, unlike helices where all amino acids show a similar pattern of periodicity of approximately of the order of the structural repeat unit of *α*-helix, i.e. 3.6 residues [40], the periodicity in propensities of the amino acids for the different inner regions with respect to the cap residues in the strands vary from one amino acid to another. This variation in periodicity may be noticed even for amino acids belonging to the same physico-chemical group. For example, the propensities of arginine are 1.07 ± 0.06, 0.86 ± 0.04, 1.13 ± 0.05, 0.84 ± 0.07 for 2nd, 4th, 6th, 8th inner positions respectively from N-cap residue, while glutamine, belonging to the same physico-chemical group exhibits propensity values of 1.02 ± 0.1, 0.78 ± 0.1, 1.06 ± 0.1 for 2nd, 5th, 8th inner positions respectively from the N-cap residue. Hence arginine shows peaks in the propensity values at a difference of four residues, while peaks in the propensity values are displayed at a difference of six residues for glutamine in the *β*-strands. In other words, the periodicity pattern of the position-specific propensities does not always follow the structural repeat of *β*-strands for all amino acids.

To validate the robustness of the propensity values of Figures 2 and 3, two additional data-sets of *β*-strands are considered, the first set consists of strands of length 5 or more residues while the second data-set comprises of strands of length ranging from 5 to 9 residues. Additional file 1, Figures S1, S2, S3 and S4 depict the position-specific propensities of the amino acids in these data-sets from both termini of *β*-strands. The results suggest similar propensity trends with respect to positions in strands as shown in figure 2 and 3. There is a good correlation between the propensity values of figure 2 and 3 (upto fifth residue from cap positions) with that of the database consisting strands of length 5 residues or more (R = 0.86 from N-Cap and R = 0.88 from C-Cap) and strands of length between 5 to 9 residues (R = 0.82 from N-Cap and R = 0.84 from C-Cap). Detailed position-wise correlation coefficients are provided in the Additional file 1, Tables S1 and S2.

It may be observed that in case of arginine and glutamine, positions of the peak propensity values as is exhibited by most of the amino acids are at differences of multiples of two, the structural repeat unit of *β*-strands. Although position-wise periodicity in *β*-strands was explained previously with binary patterning (polar-nonpolar) of amino acids [51,60] but neither the propensities of individual amino acids were considered nor the periodicity with respect to the cap positions in *β*- strands was shown. The present work with the help of considerably large database of longer strands shows that there are significant differences in the propensities of amino acids in inner positions with respect to the cap residues in strands.

Although long polar residues are considered to be unfavourable in *β*-strand structures [7,41,61], yet some of these residues show higher preferences for more than one inner positions (*P_ij _*> 1). Formation of *β*-strands even with frequent occurrences of amino acids, which have low intrinsic preferences for these structures, can be attributed to the fact that secondary structure formation is driven by the periodic occurrence of amino acids more than their intrinsic preferences [62]. As shown in Figures 2 and 3, the position-specific propensities of these residues are found to obey periodic trend though the pattern and strength of periodicity vary from residue to residue leading to the formation of *β*-strands.

Short polar amino acids exhibit a distinctly different distribution as compared to that of long polar residues. These amino acids are known to have low preferences for *β*-strands [7,41,61] and the results (Figures 2 and 3) demonstrate very weak periodic dependence of these residues with respect to the positions in *β*-strands. Asn and Asp at C-cap can form H-bond with the NH group of the residue following this position by their O*δ *atom and hence terminate *β*-sheet formation. Towards N-terminus, Asn and Asp turn the backbone of the strand preventing *β*-sheet extension from the N direction. The under-representation of aspartic acid and asparagine in the interiors of *β*-strands is due to the destabilizing effect arising from the removal of backbone-backbone H-bonding between the partner strands of *β*-sheet [48]. Among the residues from this group serine shows the strongest periodic nature in its propensity values.

Unlike the residues in polar groups, hydrophobic aromatic group amino acids are considered to be more preferred in *β*-strand structures [7,41,61]. Analysis of results confirms this trend. Yet a few inner positions of *β*-strands are under-represented by these residues e.g. N4 (= 0.70 ± 0.4), N6 (= 0.90 ± 0.4), C5 (= 0.94 ± 0.4), C7 (= 0.86 ± 0.4) positions by tryptophan, N5 (= 0.99 ± 0.1), N8 (= 0.92 ± 0.1) positions by tyrosine and C1 (= 0.86 ± 0.09), C6 (= 0.99 ± 0.09), C8 (= 0.97 ± 0.08) positions by phenylalanine. This leads to a weak periodic pattern of position-specific propensities of hydrophobic aromatic amino acids, shown in Figures 2 and 3. These amino acids usually have a very high preference for *β*-strand structures. This under-representation may be explained in terms of non-polar residue periodicity which initiates strand formation. In agreement with the earlier studies [48] a very low preference of hydrophobic aromatic amino acids is observed for both N-cap and C-cap positions.

Amino acids from aliphatic+cys group are also considered to be hydrophobic and are highly preferred in strand structures [7,41,61]. However, in contrast to hydrophobic aromatics, residues from this group show strong positional preferences in *β*-strands. For example values of cysteine starting from N1 position are 0.62 ± 0.2, 1.58 ± 0.2, 0.54 ± 0.2, 1.27 ± 0.2, 1.46 ± 0.2, 1.27 ± 0.2, 0.84 ± 0.2, 1.27 ± 0.2, 1.04 ± 0.2, 0.77 ± 0.3 upto N10 position. A similar trend of values for cysteine is also observed in the C-terminus. This position-specific periodic nature of cysteine can be rationalized based on the fact that cysteine pairs at the non-hydrogen bonded positions in antiparallel sheets favour disulphide bridge among them [63]. Methionine also exhibits a strong position-specific propensity pattern. Propensity values of methionine from N1 to N10 position are 0.84 ± 0.3, 0.91 ± 0.2, 0.67 ± 0.2, 1.25 ± 0.3, 0.88 ± 0.3, 1.18 ± 0.3, 0.84 ± 0.3, 1.14 ± 0.3, 0.88 ± 0.3, 1.11 ± 0.3. So periodicity in the position-specific propensities of methionine resembles that of the structural repeat of *β*-strands.

Analogous to helices, the amino acids belonging to other group do not show characteristic positional dependence in their propensity values in *β*-strands. Among the five residues belonging to this group only threonine is preferred in *β*-strands [7,41,61]. Results in Figures 2 and 3 show that threonine is highly preferred throughout all positions in strand structures. Another member of this group, glycine, is the only amino acid which is non-chiral. Small volume of the hydrogen in glycine imparts a local flexibility to the local peptide structure. In the present work, glycine is found to have very high preferences for both N-cap and C-cap positions (refer to Figures 2 and 3). In agreement with the earlier results, glycine is confirmed to be a strand terminator [48]. A notable difference from the earlier work is under representation of glycine in the middle region of *β*-strands. It is observed from Figures 2 and 3 that proline has higher preference for both the cap positions and is scarcely found in *β*-strands. Proline rarely fits into the regular part of helices or sheets as it lacks a NH group in the backbone for participating in H-bonding and restricted values of torsion angles [48]. The low occurrence of proline in strands is due to the fact that it has only one rotatable angle and so it loses less entropy in forming regular structures [15]. Surprisingly histidine is found to obey a periodic nature in its propensity values with respect to positions from cap residues. Previous studies have demonstrated that histidine is under-represented in the middle region of *β*-strands [48] which is in accordance with the low preference of histidine in strands [7,41,61]. However, in this work, we find quite unexpectedly that histidine is preferred in mid positions like N1 (= 1.30 ± 0.2), N3 (= 1.07 ± 0.2), N7 (= 1.17 ± 0.2) and N10 (= 1.27 ± 0.2). This together with the other positions where histidine is under-represented (*P_ij _*< 1), show a weak periodic nature in the position-specific propensity of histidine from the N-cap position. A similar trend for histidine is also found for inner positions with respect to the C-cap position.

### Propensities are independent of orientations of partner strands as well as class of proteins

Among the 1634 *β*-strands (length ≥ 10 residues) considered in this study, 1250 are found to be antiparallel, 51 are parallel while 333 of them are mixed. Additional file 1, Figures S5-S10 depict the position-specific propensities of amino acids in all three types of strands from both termini. The correlations of propensities of each amino acids in these strands with respect to the results given in the main text are shown in Additional file 1, Table S3. Excellent correlation is observed for the propensities of all amino acids between the antiparallel *β*-strands and the original database. Due to less number of parallel as well as mixed strands in the database, some positions show fluctuating propensity values. By neglecting these positions where the propensity fluctuations are greater or less than 50% of their original values, the number of amino acids which have either moderate or good correlation with the original database are found to be 13 and 19 respectively for parallel and mixed *β*-strands database.

As mentioned in the methods section an additional database of *β*-strands from *β*-barrel proteins are also considered in this study. This additional database consists of 274 *β*-strands with length ≥ 10 residues. Position-specific propensities of amino acids of these strands from both termini are shown in Additional file 1, Figures S11 and S12. A similar analysis shows that 11 amino acids have moderate or good correlation in propensity values with that of the original database. This shows that the propensity values are not biased towards any particular class of proteins. Moreover, the database comprising of 1634 *β*-strands is compiled from various proteins belonging to different classes and folds of the SCOP classified proteins [64] (class and fold annotation of these protein chains are provided in Additional file 2).

### *χ*^2^-values depict significant difference in propensities at different positions in strands

To evaluate the significance of the anisotropic propensities at different positions in strands, *χ*^2 ^values for all positions are calculated according to equation 2. All 1634 *β*-strands are included in the reference distribution for calculating these *χ*^2^-values. For a 19-dimensional system, such as amino acid distribution in different classes, *χ*^2 ^value at 95% level of confidence (probability of accepting the null hypothesis, P < 0.05) should be greater than 30.14 to reject the null hypothesis. Table 1 shows the *χ*^2 ^values for different positions in *β*-strands from both termini. Except N7, N10 and C8 (eventhough N7 position has > 90% while C8 has > 80% level of confidence), the differences in distributions of amino acids for each position in the *β*-strand are found out to be highly significant. *χ*^2 ^values are also calculated by considering the 1634 *β*-strand sequences excluding the cap positions. The results are shown in parenthesis of Table 1 for N1 to N10 and C1 to C10 positions. The trend in *χ*^2 ^values are more or less similar to that obtained by including the cap positions.

**Table 1:** *χ*^2 ^values for Amino Acid Compositions at different positions in *β*-strands.

Positions from N-terminal	***χ***^**2**^	Positions from C-terminal	***χ***^**2**^
N-cap	631.1	C-cap	464.1
N1	69.9 (67.7)	C1	140.2(177.0)
N2	89.9(69.0)	C2	62.8(42.9)
N3	99.8(74.1)	C3	59.3(39.6)
N4	54.5 (50.4)	C4	76.3(51.9)
N5	39.6(34.2)	C5	54.5(38.2)
N6	71.4(48.3)	C6	36.3(25.4)
N7	26.9(17.5)	C7	47.9(36.5)
N8	51.1(38.9)	C8	23.8(15.3)
N9	37.9(21.1)	C9	53.4(37.5)
N10	17.0(18.8)	C10	52.2(40.1)

Data in parenthesis show *χ*^2 ^values calculated by neglecting the cap residues.

Periodic propensity values are not an artefact of the amino acid composition in *β*-strands. To verify this a random scrambling of the 1634 *β*-strand sequences (including the cap residues) are done to get 1634 random peptide sequences. Position-specific propensities of amino acids in these random peptide sequences from both the termini are shown in Additional file 1, Figures S13 and S14. It may be observed that the position-specific propensity curves are almost flat, without any periodicity in the peak propensity values. The featureless curves of the random peptide sequences lack marked periodicity especially when the propensities of the amino acids are observed with respect to the C-terminus. Hence it may be emphasized that the periodicity is position-specific and not a consequence of the amino acid composition of *β*-strands.

### Average hydrophobicity is periodic in nature from cap position in *β*-strands

**Figure 4:**
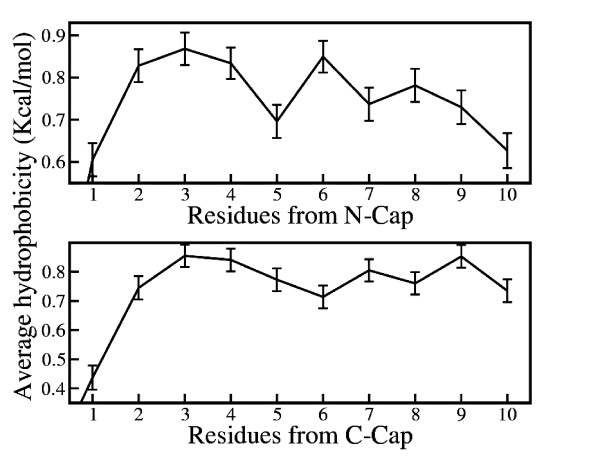
**Position-Specific Average Hydrophobicity**. Average hydrophobicity plotted against position from the cap residues in strands. Position-specific hydrophobicity for each *β*-strands are calculated and standard error of the data are plotted as error bars.

The propensity results suggest that both the polar and the hydrophobic residues have specific positional preference to occur in *β*-strands. To explore further, position-specific average hydrophobicity is calculated for 10 inner positions from both termini of *β*-strands according to equation 4. Figure 4 illustrates this average hydrophobicity plotted vs. positions in *β*-strands. The graphs show average hydrophobicity is periodic in nature with respect to positions in *β*-strands. The periodicity of average hydrophobicity with respect to positions in secondary structures has been observed earlier and is thought to play an important role in stabilizing these structures [53,65]. However, it was not investigated in relation to the cap position in *β*-strands. Present investigation with the help of a much larger database shows that average hydrophobicity is periodic in nature in inner positions with respect to cap residues in strands. A similar result is obtained in case of helices earlier [40]. The position-specific periodic nature of average hydrophobicity is different in the strength of periodicity for *β*-strands as compared to helices. While for helices position-specific average hydrophobicity ranges between around 0.2 to 0.8 Kcal/mol, the range in case of strands is between 0.6 to 0.8 Kcal/mol. Very low hydrophobicity of N-cap (0.08 Kcal/mol) and C-cap (0.1 Kcal/mol) residues also confirm the presence of a hydrophilic barrier at both termini of *β*-strands (data not shown in Figure 4).

### Free energy values are different at different positions

The *χ*^2 ^values from equation 2 clearly indicate that inner positions from both termini of *β*-strands have their intrinsic characteristic amino acid requirements. Differences in the amino acid propensities at each of these 10 positions are markedly pronounced, especially at the cap positions and in the middle positions, i.e., across N4-N10 at the N-terminus and C3-C8 at the C-terminus. Most of the propensity scales reflect the free energy differences of the different amino acid residues in the respective sequence positions. This free energy difference is calculated by equation 5. The free energy values (Additional file 1, Tables S4 and S5) clearly distinguish the specific positional preferences of the respective amino acids, which may be used for designing *β*-strands. In agreement with the previous studies [41] Ala is found to be more stable than Gly in sheets. The position-specific propensities of amino acids are found to correlate well with position-specific hydrophobicities. The position-specific propensity of valine is very high and is directly proportional to that of position-specific hydrophobicity at both N- and C-terminus with correlation coefficient of 0.96 and 0.92 respectively. For proline, the hydrophobicity trend is exactly opposite in keeping with the values of position-specific propensity (R = -0.85 and -0.87 respectively from N- and C-terminus). In general, the hydrophobic amino acids have a better correlation of their propensity values to the position-specific hydrophobicity in comparison to the polar ones (Additional file 1, Table S6). This trend in position-specific propensity and the correlation between its values with position-specific hydrophobicity can be a suitable input for secondary structure prediction algorithms and *de novo *protein design.

## Conclusions

In contrast to the earlier findings, amino acid propensities are found to be position-specific throughout *β*-strands. Periodicity plays the role of an important stabilizing factor for the secondary structures, especially for *α*-helices [40]. This work, for the first time, presents a detailed analysis of the position-specific propensities of amino acids in *β*-strands with a large database of non-redundant proteins. Analogous to the *α*-helices, the position-specific propensities of amino acids in *β*-strands are found to exhibit an unusual characteristic periodic behavior with respect to the cap residues of *β*-strands in both termini. This periodic nature is different for different amino acids, even amino acids belonging to same physico-chemical groups display different patterns in their position-specific propensity. In a nutshell amino acids belonging to aliphatics+cys group (particularly cysteine and methionine) show strong periodicity in their propensity values; long polar (particularly arginine and glutamine) and hydrophobic aromatic (particularly phenylalanine) group amino acids show very mild periodicity in their position-specific propensity values; while no periodic pattern is found for amino acids belonging to short polar and other group. The position-dependence of these residues may be attributed due to the fact that different residues (e.g. polar, aromatic etc.) may have different tendencies to appear inside the protein core as opposed to the surface. The positions of the peak values of propensity displayed by most of the amino acids are at differences of multiples of two, the structural repeat unit of *β*-strands. Average hydrophobicity also shows position-specific periodic nature in strands. The physico-chemical characteristics of the amino acids combined with the position-specific propensity and hydrophobicity measures may direct de novo designing of proteins, primarily comprising of *β*-strands (*β*-sheet proteins), whose structures are difficult to determine [66].

## Authors' contributions

NB and PB have designed research. NB has performed research. PB and NB have analyzed data, written the manuscript and approved the final version.

## Supplementary Material

Additional file 1 **Position-Specific Propensities for Smaller *β*-strands, Position-Specific Free Energy of Amino Acids and Correlation Tables**. The file contains figures consisting of position-specific propensities of amino acids in *β*-strands of length ≥ 5 residues and 5 to 9 residues (Additional Figures S1, S2, S3 and S4). It also provides the correlation values between these position-specific propensities and those found in the main text (Additional Tables S1 and S2). Additional Tables S4 and S5 provide the position-specific free energies of the 20 amino acids from both N- and C-terminus. Correlation values between position-specific propensities and average hydrophobicity are also provided in this file (Additional Table S6). Click here for file

Additional file 2 **Class and fold annotation from SCOP classification**. The file tabulates the class and fold annotation of protein chains from which the 1634 *β*-strands analysed in this study are taken. Click here for file
