# Supplementary material for: Position-specific propensities of amino acids in the β-strand
Source: BMC Struct Biol. 2010 Sep 28;10:29. doi: 10.1186/1472-6807-10-29 (PMC2955036; doi:10.1186/1472-6807-10-29)
Supplement: Additional file 1 — Position-Specific Propensities for Smaller β-strands, Position-Specific Free Energy of Amino Acids and Correlation Tables. The file contains figures consisting of position-specific propensities of amino acids in β-strands of length ≥ 5 residues and 5 to 9 residues (Additional Figures S1, S2, S3 and S4). It also provides the correlation values between these position-specific propensities and those found in the main text (Additional Tables S1 and S2). Additional Tables S4 and S5 provide the position-specific free energies of the 20 amino acids from both N- and C-terminus. Correlation values between position-specific propensities and average hydrophobicity are also provided in this file (Additional Table S6). [file 1472-6807-10-29-S1.PDF]

Additional figure S1

Position specific propensities of amino acids up to fifth inner positions from N-Cap of β-strands. The data base consists of β-strands of length 5 residues or longer.

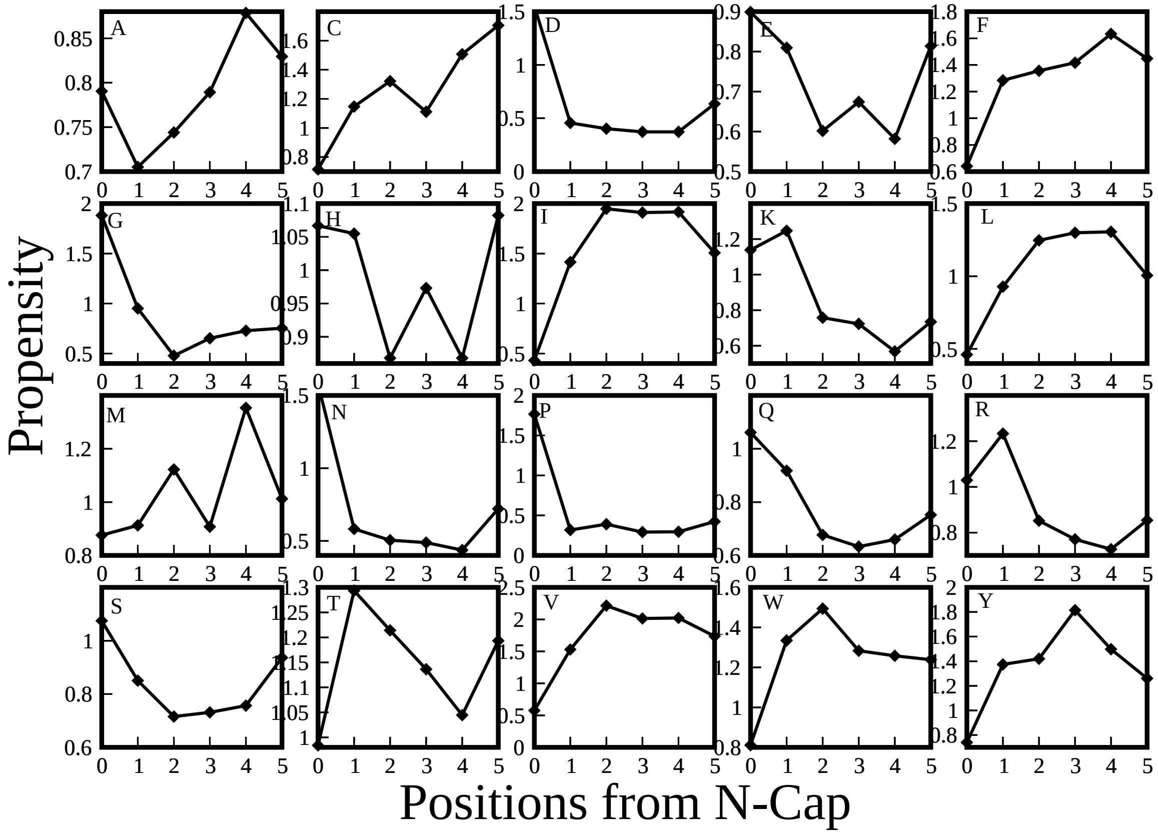

Additional figure S2

Position specific propensities of amino acids up to fifth inner positions from C-Cap of β-strands. The data base consists of β-strands of length 5 residues or longer.

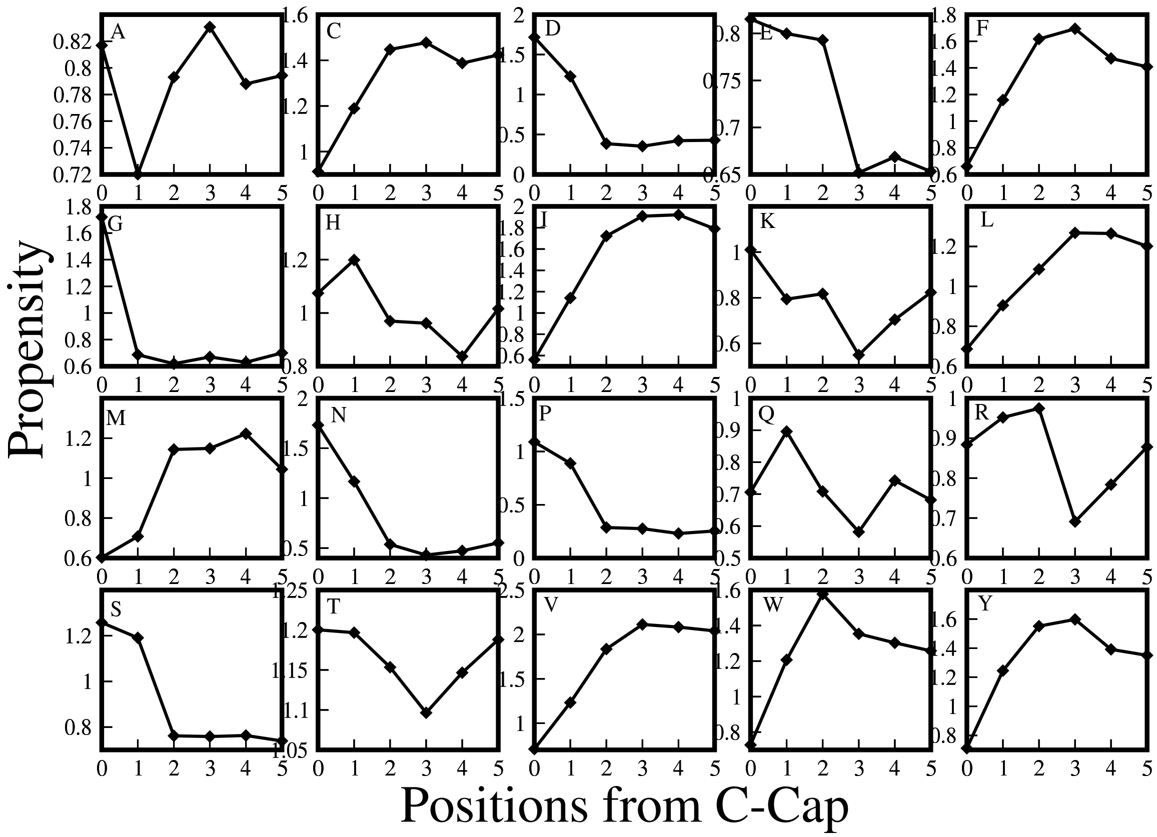

Additional figure S3

Position specific propensities of amino acids up to fifth inner positions from N-Cap of β-strands. The data base consists of β-strands of length 5 to 9 residues long.

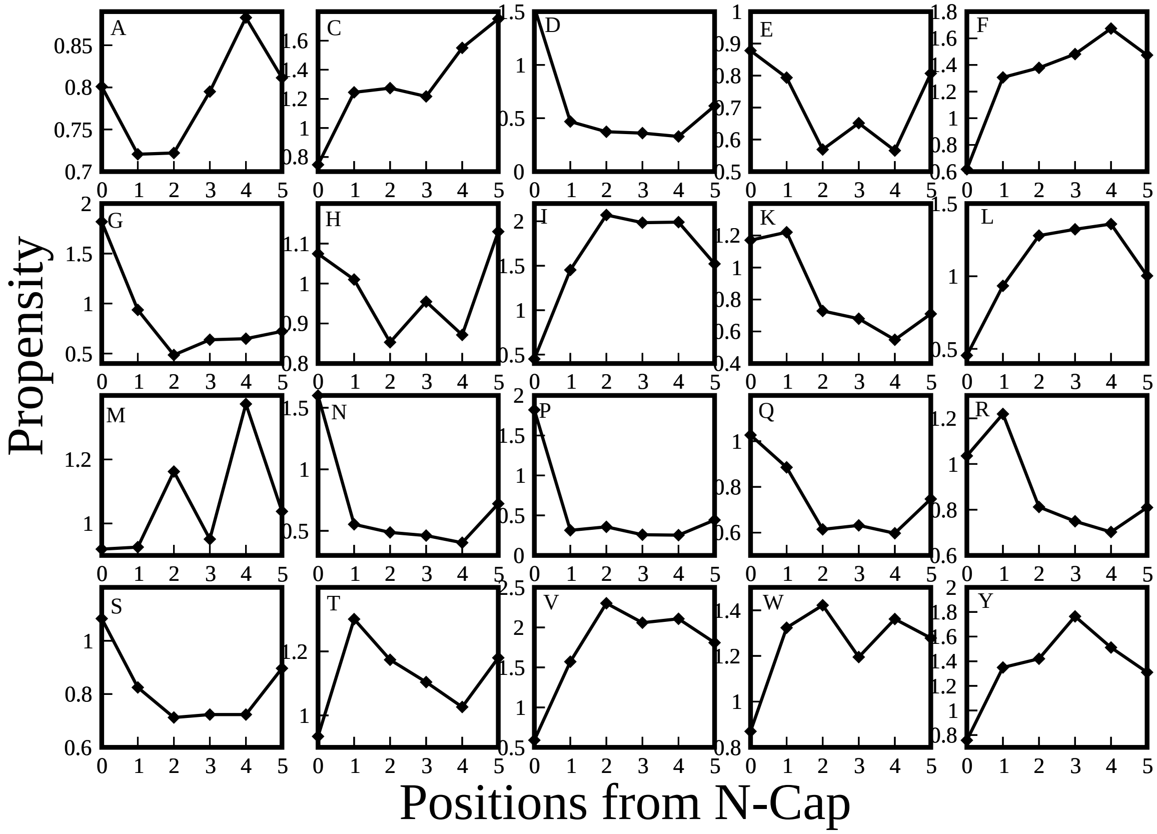

Additional figure S4

Position specific propensities of amino acids up to fifth inner positions from C-Cap of β-strands. The data base consists of β-strands of length 5 to 9 residues long.

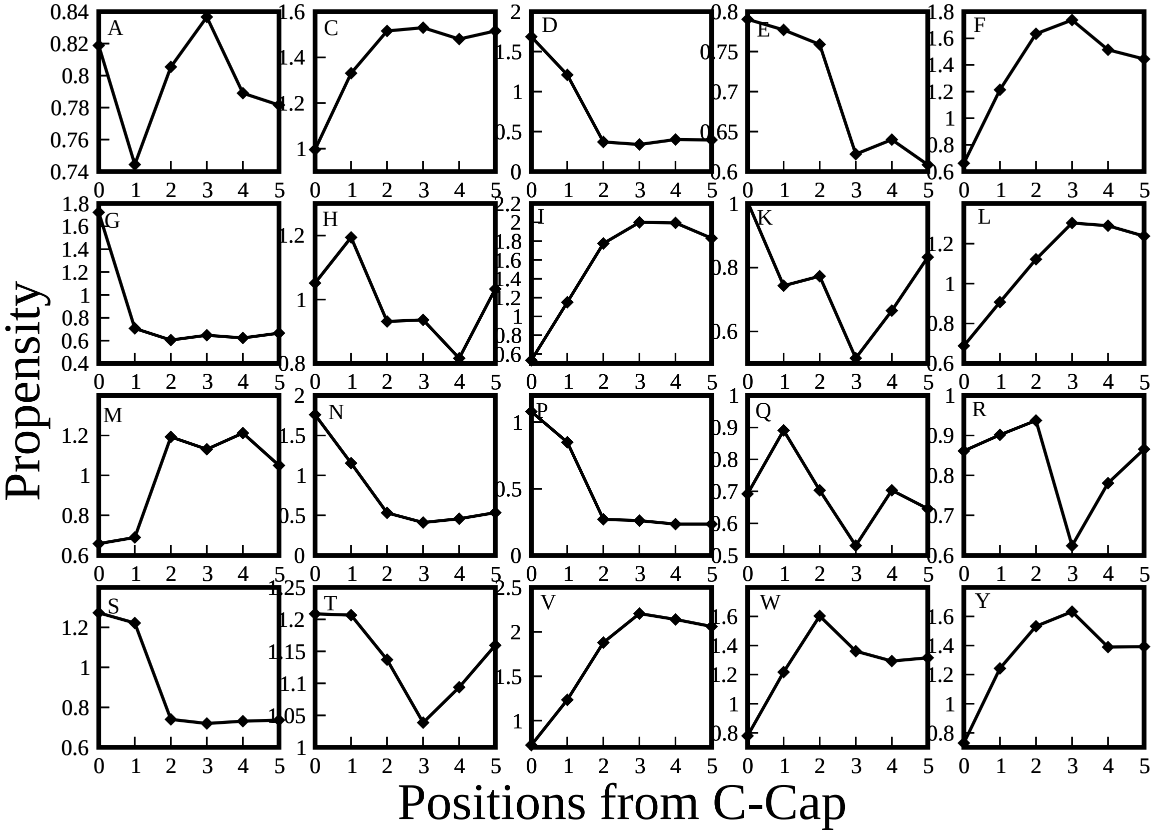

Additional table S1

Correlation coefficient between position specific propensity values from original dataset of 10 residues or longer strands (10>) with that from the additional two datasets first 5 residues or longer strands (5>) and second strands between 5 to 9 residue long. The table is for propensities from N terminus.

Positions	10> vs 5>	10> vs 5 to 9
N-cap	0.93	0.90
N1	0.86	0.81
N2	0.86	0.82
N3	0.86	0.81
N4	0.88	0.86
N5	0.88	0.84
Total	0.86	0.82

Additional table S2

Correlation coefficient between position specific propensity values from original dataset of 10 residues or longer strands (10>) with that from the additional two datasets first 5 residues or longer strands (5>) and second strands between 5 to 9 residue long. The table is for propensities from C terminus.

Positions	10> vs 5>	10> vs 5 to 9
Ccap	0.92	0.89
C1	0.53	0.37
C2	0.92	0.90
C3	0.92	0.90
C4	0.91	0.89
C5	0.91	0.89
Total	0.88	0.84

Additional figure S5

Position specific propensities of amino acids in antiparallel β-strands from N-terminus. The range of y-axis is similar to Figure 2 of main text.

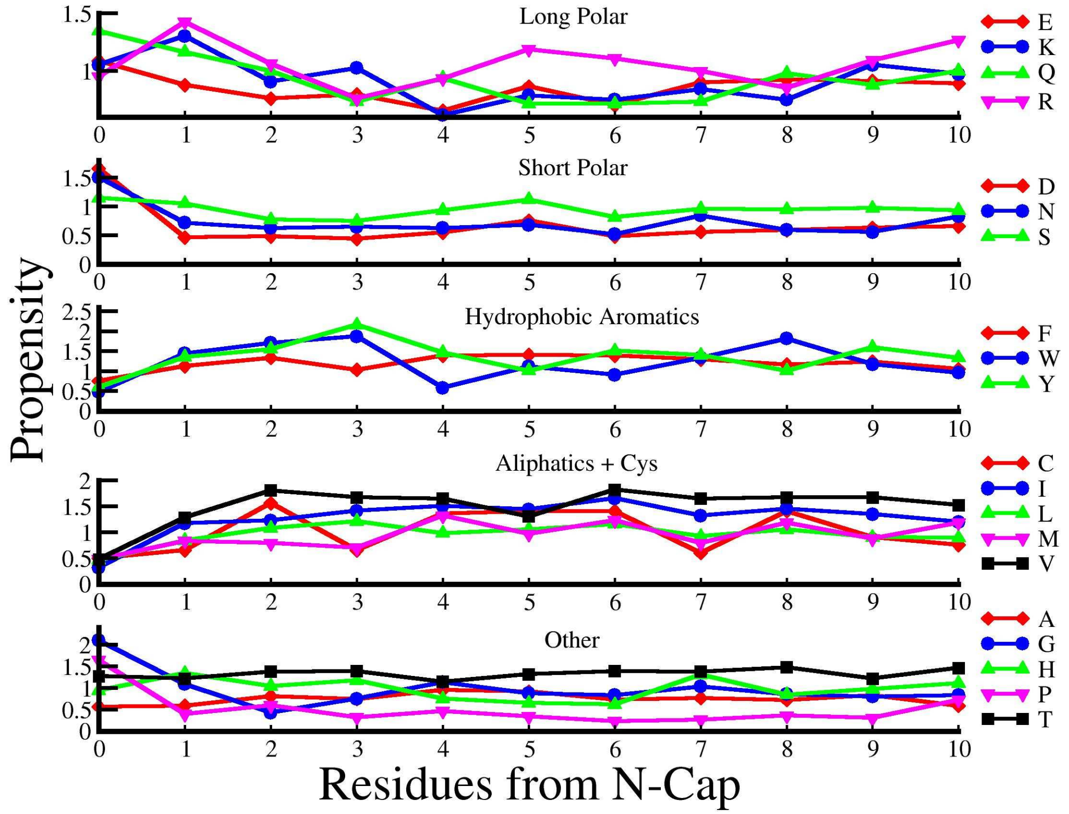

Additional figure S6

Position specific propensities of amino acids in antiparallel β-strands from C-terminus. The range of y-axis is similar to Figure 3 of main text.

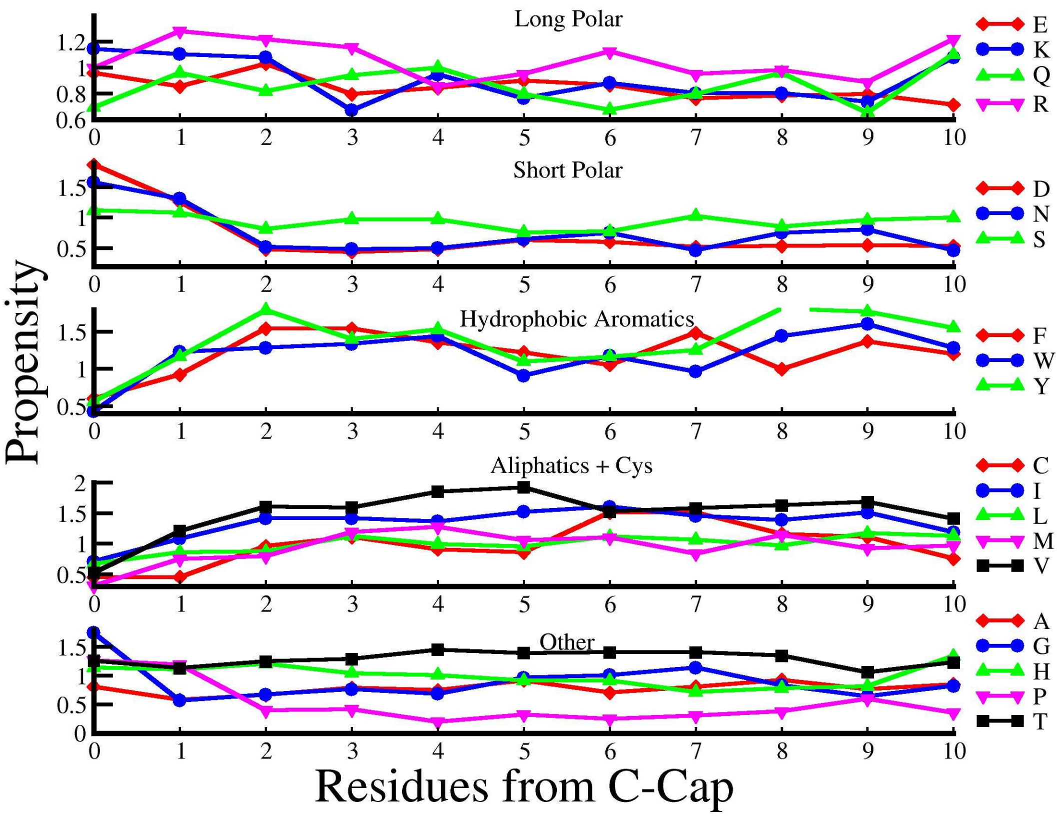

Additional figure S7

Position specific propensities of amino acids in parallel β-strands from N-terminus. The range of y-axis is similar to Figure 2 of main text.

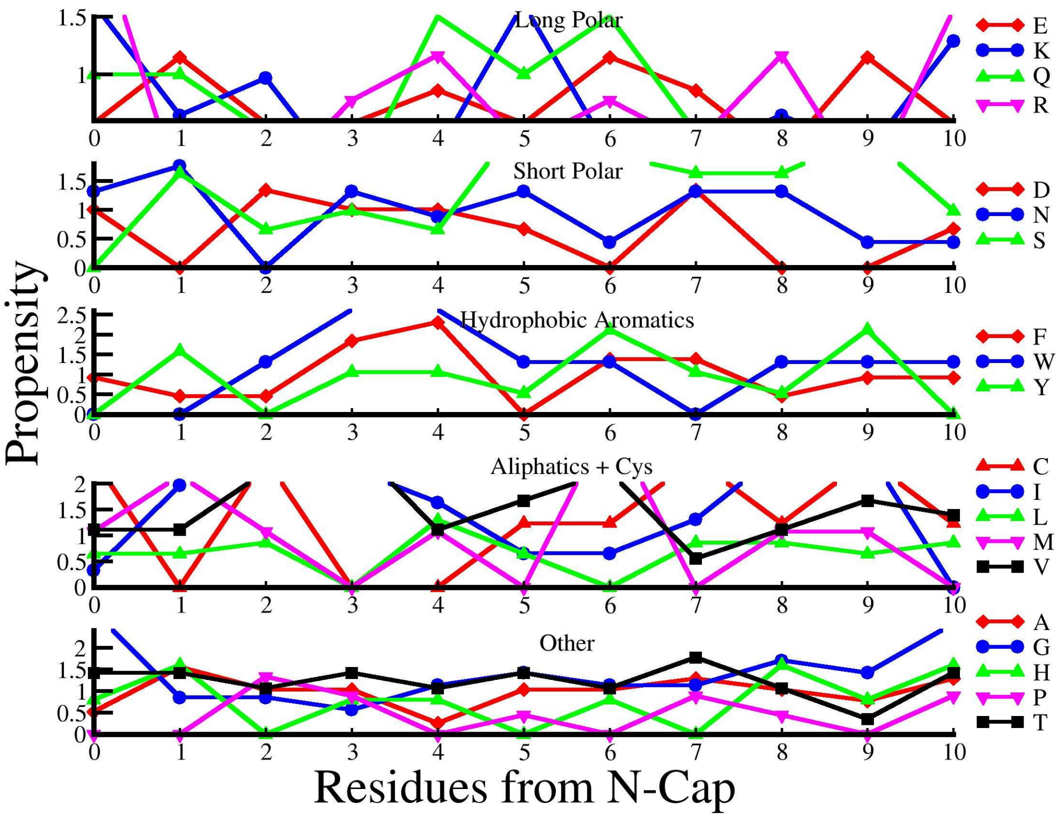

Additional figure S8

Position specific propensities of amino acids in parallel β-strands from C-terminus. The range of y-axis is similar to Figure 3 of main text.

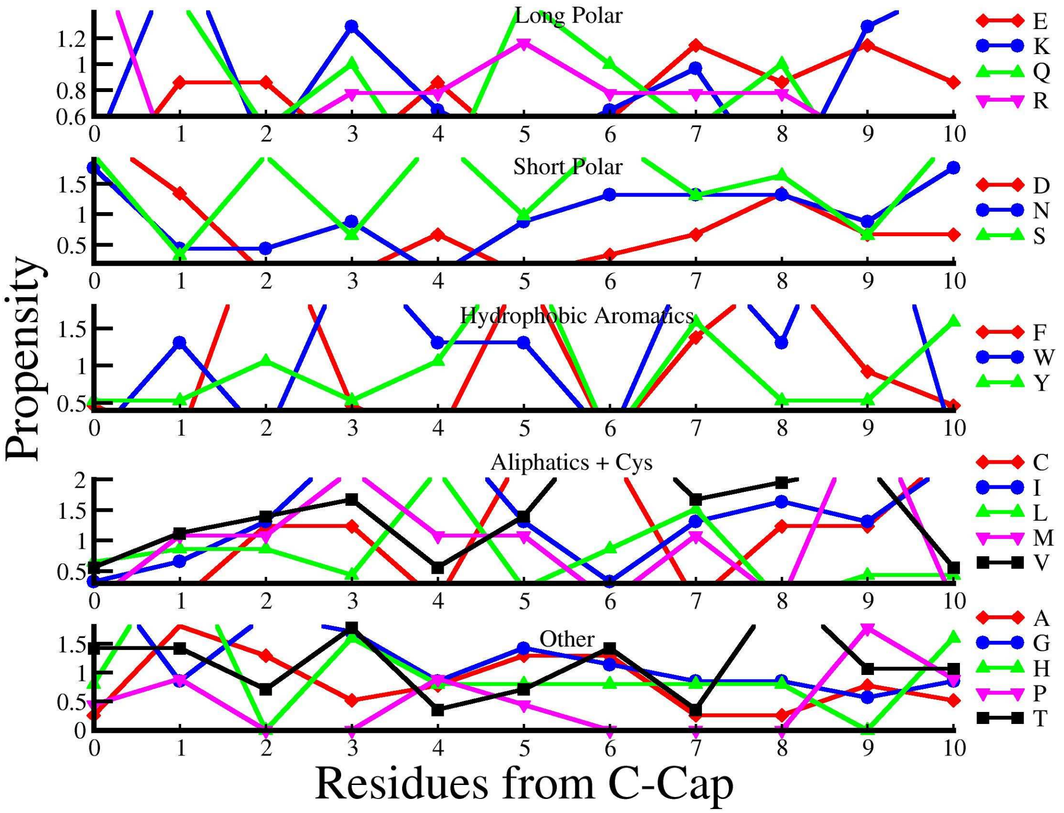

Additional figure S9

Position specific propensities of amino acids in mixed β-strands from N-terminus. The range of y-axis is similar to Figure 2 of main text.

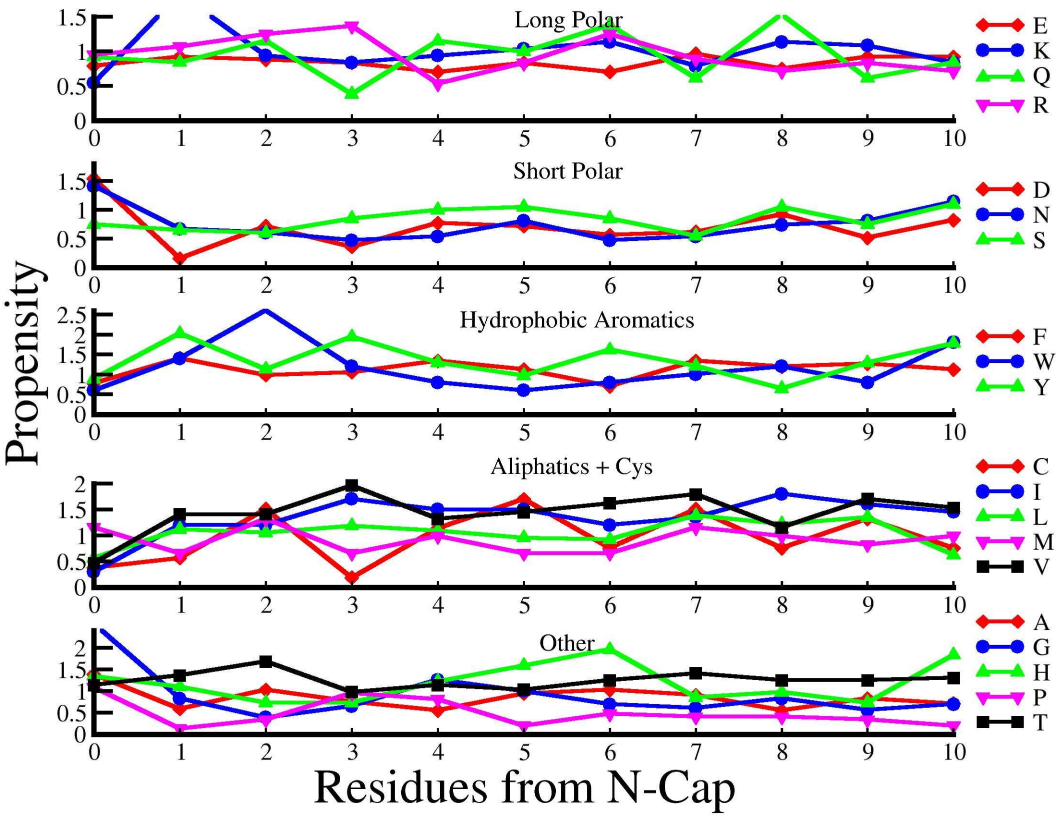

Additional figure S10

Position specific propensities of amino acids in mixed β-strands from C-terminus. The range of y-axis is similar to Figure 3 of main text.

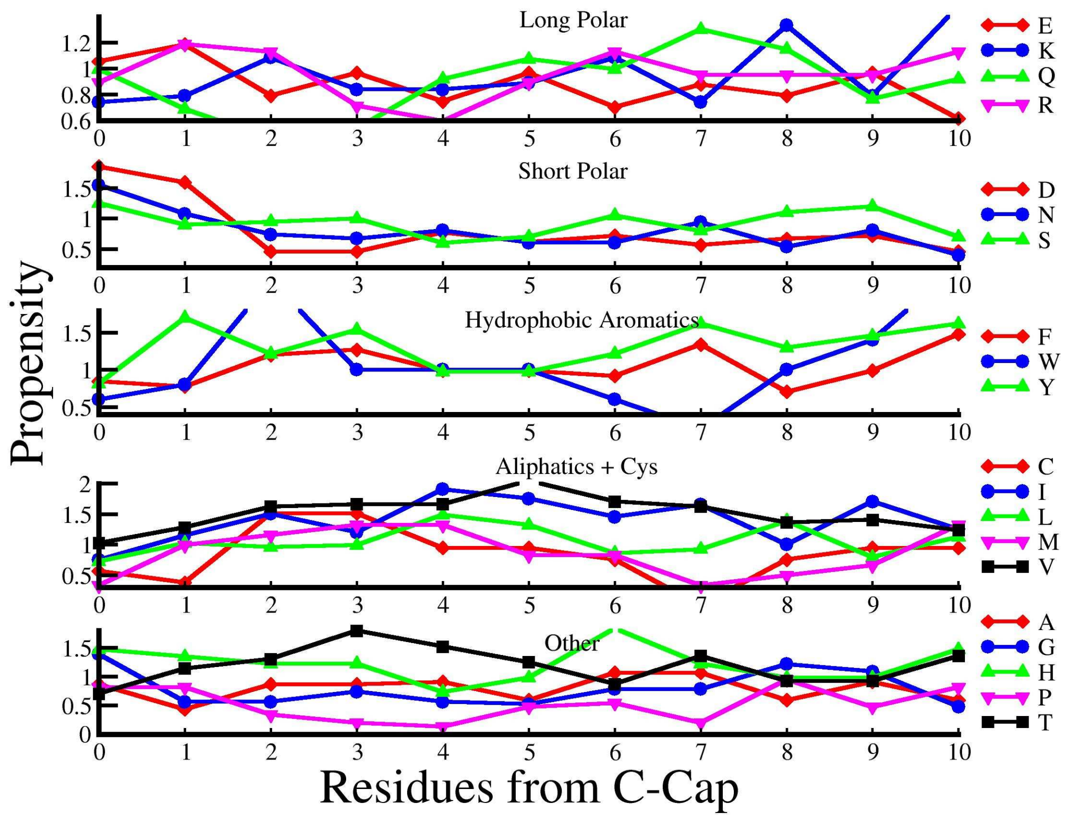

Additional figure S11

Position specific propensities of amino acids in β-strands of beta-barrel proteins from N-terminus. The range of y-axis is similar to Figure 2 of main text.

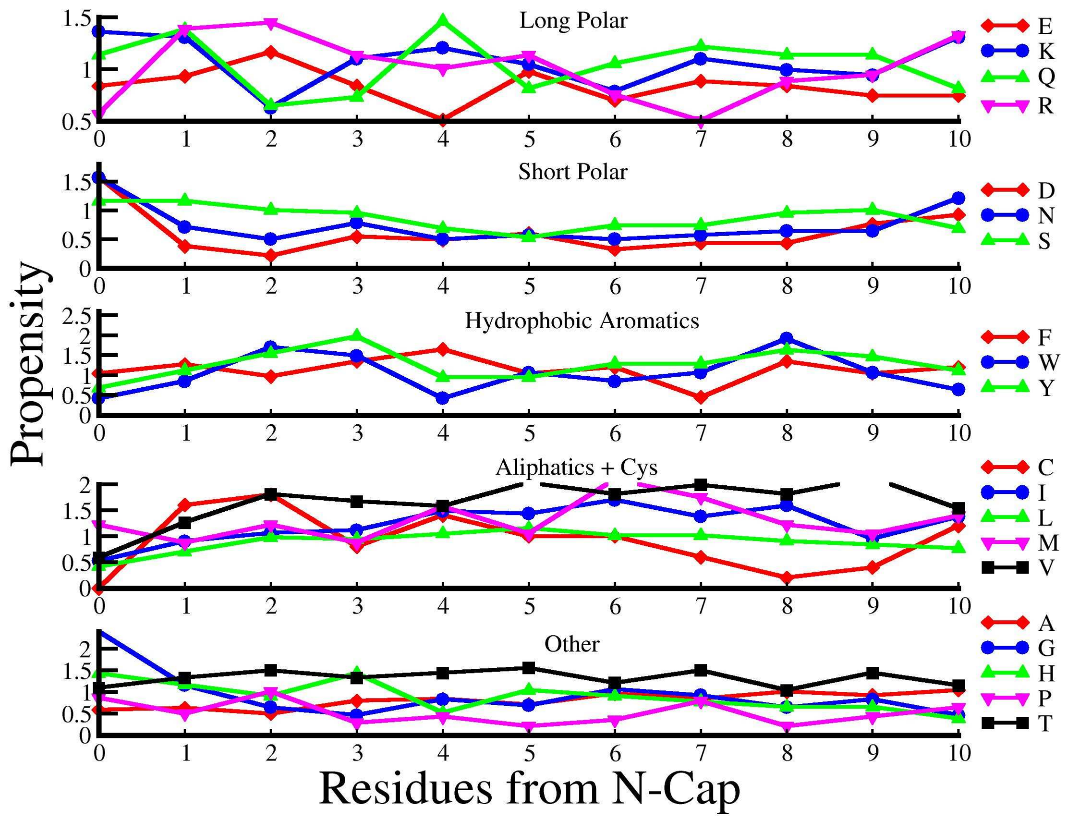

Additional figure S12

Position specific propensities of amino acids in β-strands of beta-barrel proteins from C-terminus. The range of y-axis is similar to Figure 3 of main text.

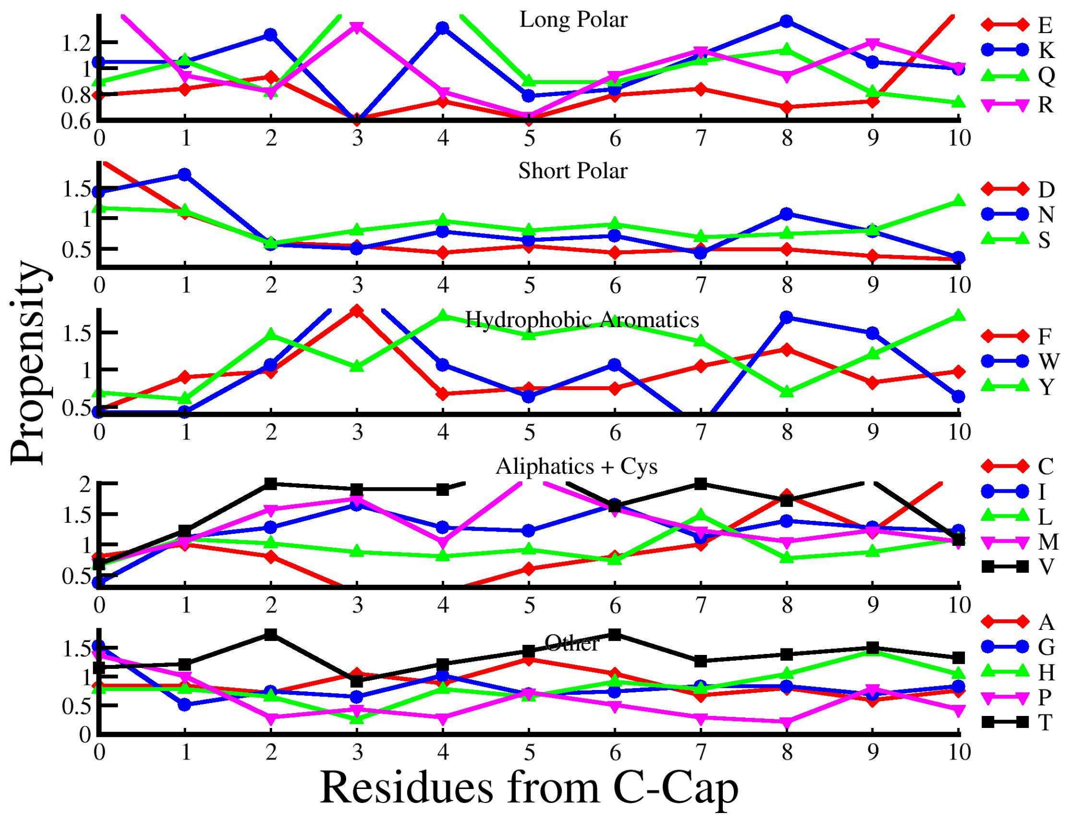

Additional table S3

The correlation coefficients of the position specific propensities of the amino acids with that of the results given in main manuscript (Figure 2 and 3). Due to the less number of parallel, mixed β-strands and beta strands from beta-barrel proteins some positions show highly fluctuating propensity values. The correlation coefficients are calculated by neglecting these values corresponding to such positions. The number of positions (out of 11) which are considered are given in the parenthesis.

Main	Antiparallel		Parallel		Mixed		Beta-barrel	
A. Acid	N-term	C-term	N-term	C-term	N-term	C-term	N-term	C-term
A	0.87	0.86	0.30 (7)	0.87 (5)	0.59 (10)	0.40 (11)	.003 (10)	-.05 (10)
C	0.96	0.93	0 (3)	0 (4)	0.79 (9)	0.59 (10)	0.73 (6)	0.18 (5)
D	0.99	0.99	0.99 (3)	0.96 (7)	0.93 (10)	0.98 (11)	0.91 (10)	0.96 (11)
E	0.97	0.91	0.10 (9)	-.05 (8)	0.56 (11)	0.61 (11)	0.31 (11)	0.45 (10)
F	0.96	0.99	0.64 (5)	0.95 (3)	0.49 (11)	0.73 (11)	0.32 (10)	0.50 (11)
G	0.99	0.98	0.96 (6)	0.40 (7)	0.97 (11)	0.70 (11)	0.90 (11)	0.94 (10)
H	0.86	0.93	0.91 (7)	0.64 (8)	0.41 (9)	0.75 (10)	0.44 (10)	0.11 (9)
I	0.98	0.97	0.99 (3)	0.63 (6)	0.91 (11)	0.84 (11)	0.54 (10)	0.78 (11)
K	0.95	0.94	0.98 (3)	0.76 (4)	0.63 (11)	0.58 (10)	0.45 (10)	0.45 (10)
L	0.96	0.90	0.35 (9)	0.69 (5)	0.69 (11)	0.46 (11)	0.85 (11)	0.21 (11)
M	0.97	0.96	0 (4)	0 (5)	0.19 (10)	0.70 (10)	0.91 (8)	0.48 (6)
N	0.99	0.99	0.78 (5)	0.97 (4)	0.85 (11)	0.90 (10)	0.90 (11)	0.87 (11)
P	0.99	0.99	0.98 (3)	1 (2)	0.95 (6)	0.95 (8)	0.86 (8)	0.96 (8)
Q	0.91	0.88	0.67 (5)	0.29 (5)	0.62 (10)	0.35 (11)	0.48 (10)	0.34 (9)
R	0.94	0.98	0.24 (5)	-.12 (6)	0.55 (10)	0.79 (11)	0.46 (11)	0.01 (10)
S	0.91	0.90	0.18 (4)	-.15 (4)	0.44 (11)	0.48 (11)	0.18 (10)	0.52 (11)
T	0.92	0.87	0.05 (10)	0.14 (8)	0.52 (11)	0.67 (11)	-.29 (11)	-.26 (11)
V	0.99	0.99	0.68 (8)	0.74 (8)	0.86 (11)	0.85 (10)	0.91 (10)	0.92 (11)
W	0.97	0.95	0.44 (7)	0 (4)	0.77 (10)	0.81 (8)	0.88 (11)	0.83 (7)
Y	0.98	0.98	0.51 (8)	0.71 (5)	0.78 (11)	0.52 (11)	0.87 (10)	0.53 (9)

Additional figure S13

Position specific propensities of amino acids in 1634 peptide sequences created by randomizing β-strands studied in the main manuscript from N-terminus. The range of y-axis is similar to Figure 2 of main text.

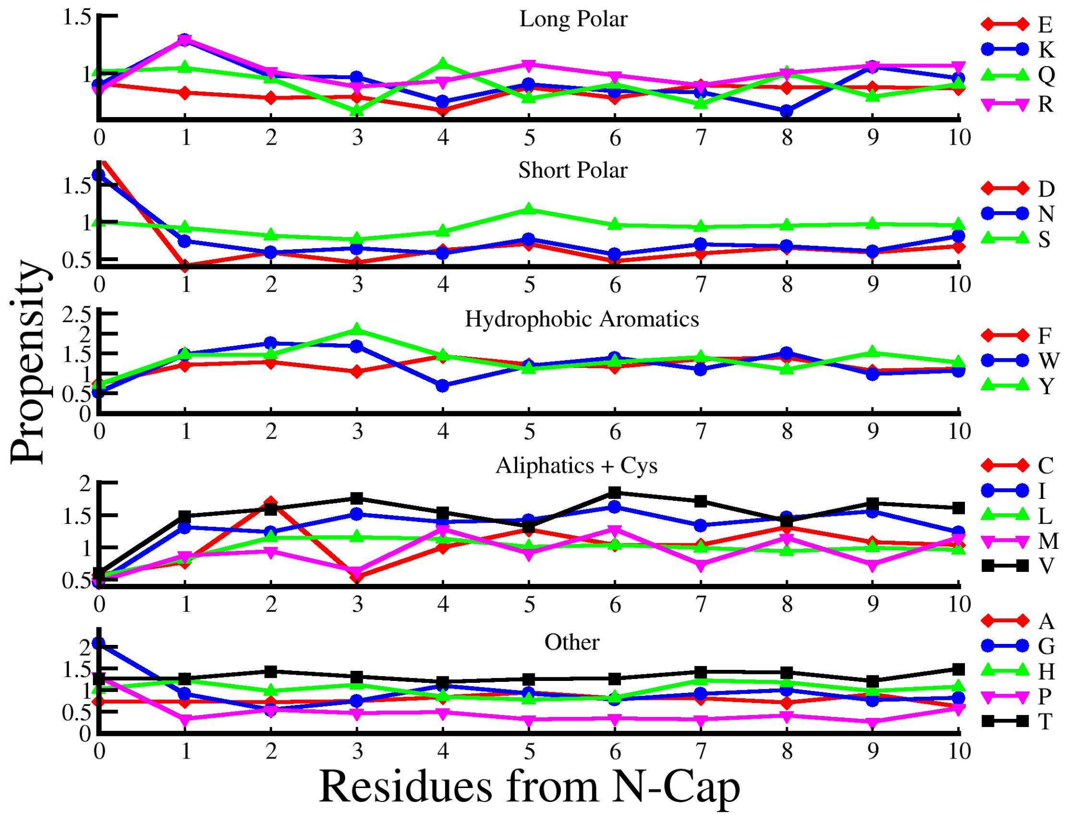

Additional figure S14

Position specific propensities of amino acids in 1634 peptide sequences created by randomizing β-strands studied in the main manuscript from C-terminus. The range of y-axis is similar to Figure 3 of main text.

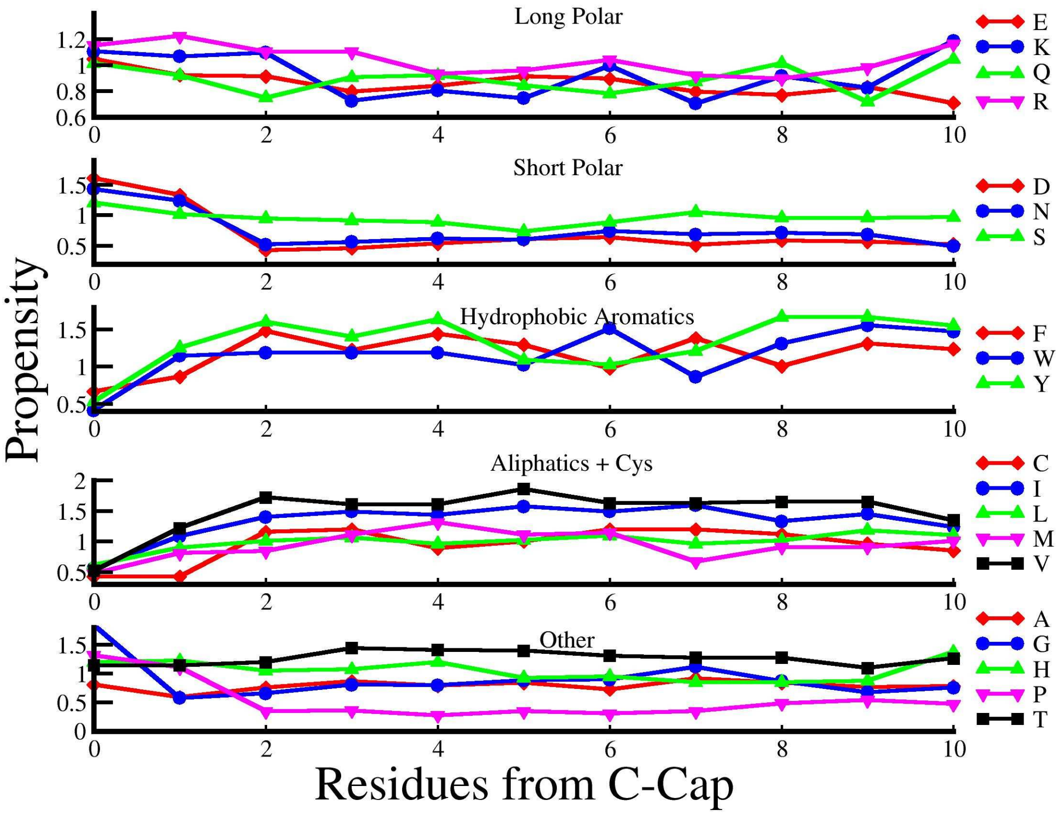

Additional table S4

Free energy of the amino acids from N terminus calculated by equation 7.

AA	NC	N1	N2	N3	N4	N5	N6	N7	N8	N9	N10
A	0.45	0.69	0.21	0.40	0.23	0.11	0.31	0.30	0.51	0.27	0.65
V	1.01	-0.38	-0.80	-0.82	-0.65	-0.43	-0.84	-0.72	-0.63	-0.75	-0.61
L	1.03	0.16	-0.10	-0.23	-0.02	-0.03	-0.11	-0.02	-0.12	0.02	0.25
I	1.66	-0.27	-0.35	-0.59	-0.60	-0.52	-0.62	-0.41	-0.65	-0.53	-0.28
P	-0.57	1.59	0.82	1.09	0.93	1.65	1.85	1.65	1.42	1.71	0.68
M	0.64	0.25	0.14	0.57	-0.32	0.19	-0.24	0.25	-0.19	0.19	-0.15
F	0.39	-0.22	-0.31	-0.09	-0.50	-0.39	-0.33	-0.39	-0.21	-0.31	-0.09
W	1.02	-0.48	-0.91	-0.82	0.52	-0.03	0.15	-0.30	-0.75	-0.15	-0.20
G	-1.15	-0.04	1.20	0.46	-0.22	0.11	0.29	0.07	0.19	0.37	0.21
S	-0.04	0.02	0.45	0.37	0.09	-0.21	0.22	0.16	0.02	0.05	0.05
T	-0.33	-0.33	-0.52	-0.39	-0.19	-0.34	-0.44	-0.48	-0.51	-0.26	-0.52
C	0.89	0.70	-0.66	0.89	-0.35	-0.55	-0.35	0.24	-0.35	-0.06	0.38
N	-0.57	0.43	0.73	0.66	0.69	0.46	0.98	0.33	0.63	0.73	0.19
Q	-0.32	-0.13	-0.02	0.64	0.00	0.36	0.19	0.51	-0.09	0.30	0.09
Y	0.63	-0.59	-0.51	-1.06	-0.51	0.01	-0.64	-0.44	0.11	-0.64	-0.47
D	-0.69	1.37	0.85	1.18	0.72	0.43	1.05	0.74	0.64	0.77	0.53
E	-0.02	0.16	0.36	0.33	0.57	0.23	0.48	0.13	0.20	0.12	0.17
K	0.05	-0.47	0.13	0.06	0.57	0.21	0.29	0.29	0.28	-0.05	0.06
R	0.01	-0.39	-0.09	0.18	0.22	-0.13	-0.17	0.06	0.26	-0.01	-0.22
H	-0.03	-0.38	0.08	-0.10	0.24	0.28	0.15	-0.23	0.15	0.11	-0.35

Additional table S5

Free energy of the amino acids from C terminus calculated by equation 7.

AA	CC	C1	C2	C3	C4	C5	C6	C7	C8	C9	C10
A	0.31	0.76	0.46	0.32	0.35	0.21	0.32	0.24	0.25	0.32	0.34
V	0.67	-0.28	-0.68	-0.69	-0.83	-0.95	-0.69	-0.67	-0.66	-0.72	-0.43
L	0.56	0.16	0.17	-0.11	-0.18	0.00	-0.08	-0.07	-0.03	-0.10	-0.14
I	0.50	-0.12	-0.52	-0.50	-0.61	-0.65	-0.62	-0.58	-0.40	-0.63	-0.31
P	-0.20	-0.15	1.42	1.47	2.27	1.47	1.71	1.85	1.04	0.71	1.09
M	1.72	0.31	0.19	-0.32	-0.36	-0.01	-0.01	0.43	0.03	0.09	-0.01
F	0.62	0.21	-0.61	-0.54	-0.31	-0.28	0.01	-0.54	0.03	-0.36	-0.31
W	1.15	-0.20	-0.52	-0.39	-0.44	0.09	-0.03	0.22	-0.44	-0.71	-0.56
G	-0.77	0.79	0.53	0.34	0.59	0.17	0.05	-0.08	0.13	0.46	0.41
S	-0.23	-0.03	0.19	0.05	0.09	0.41	0.19	0.02	0.11	0.00	0.03
T	-0.21	-0.19	-0.31	-0.50	-0.52	-0.43	-0.38	-0.45	-0.38	-0.05	-0.33
C	1.11	1.24	-0.11	-0.26	0.17	0.11	-0.47	-0.21	-0.11	-0.11	0.24
N	-0.66	-0.31	0.83	0.90	0.86	0.63	0.43	0.76	0.46	0.30	1.02
Q	0.36	0.12	0.45	0.22	0.07	0.19	0.42	0.17	0.00	0.61	-0.07
Y	0.71	-0.33	-0.73	-0.49	-0.49	-0.17	-0.19	-0.42	-0.74	-0.74	-0.65
D	-0.91	-0.41	1.12	1.22	0.87	0.72	0.69	0.90	0.77	0.77	0.93
E	0.07	0.12	0.03	0.29	0.28	0.16	0.28	0.33	0.34	0.25	0.52
K	-0.05	-0.09	-0.08	0.46	0.13	0.37	0.13	0.33	0.17	0.39	-0.24
R	-0.01	-0.29	-0.23	-0.08	0.32	0.08	-0.16	0.08	0.04	0.18	-0.22
H	-0.26	-0.29	-0.23	-0.14	0.08	0.11	-0.14	0.28	0.28	0.28	-0.46

Additional table S6

Correlation of the position wise propensities of the amino acid groups with that of position wise hydrophobicity.

Amino Acid Types	N-term	C-term
Long Polar	0.62(-)	0.56(-)
Short Polar	0.92(-)	0.94(-)
Hydrophobic	0.98	0.98
Other	0.91(-)	0.79(-)
