# Supplementary material for: Position-specific propensities of amino acids in the β-strand
Source: BMC Struct Biol. 2010 Sep 28;10:29. doi: 10.1186/1472-6807-10-29 (PMC2955036; doi:10.1186/1472-6807-10-29)
Supplement: Additional file 2 — Class and fold annotation from SCOP classification. The file tabulates the class and fold annotation of protein chains from which the 1634 β-strands analysed in this study are taken. [file 1472-6807-10-29-S2.PDF]

```
******************************************************************************
******************************************************************************
******************************************************************************
***********       ********        ********        ********        ************
***********  *************  **************  ****  ********  ****  ************
***********  *************  **************  ****  ********  ****  ************
***********  *************  **************  ****  ********  ****  ************
***********       ********  **************  ****  ********        ************
****************  ********  **************  ****  ********  ******************
****************  ********  **************  ****  ********  ******************
****************  ********  **************  ****  ********  ******************
***********       ********        ********        ********  ******************
******************************************************************************
******************************************************************************
******************************************************************************
```

SCOP

Out of the 774 protein chains containing 1634 beta-sheets studied in the main text, 15 belong to All alpha proteins class, 199 belong to All beta proteins class, 146 belong to Alpha and beta proteins (a+b) class, 60 belong to Alpha and beta proteins (a/b) class, 4 belong to Small proteins class, 4 belong to Membrane & cell surface class and 7 belong to Multi-domain proteins class of SCOP. 339 protein chains are not classified by SCOP. These protein chains belong to 270 different folds of SCOP. The SCOP classes and folds of the protein chains are tabulated below.

PDB	CLASS	FOLD
1RHI1	All beta proteins	Nucleoplasmin-like/VP (viral c oat and capsid proteins)
1GQWB	All beta proteins	Double-stranded beta-helix
1WC4B	Alpha and beta proteins (a+b)	Ferredoxin-like
2BHVE		
1FL7D	Small proteins	Cystine-knot cytokines
1C4TB	Alpha and beta proteins (a/b)	CoA-dependent acyltransferases
1UK1A	All alpha proteins	Domain of poly(ADP-ribose) pol ymerase ADP-ribosylation
1QC9A	Alpha and beta proteins (a/b)	Restriction endonuclease-like
1T8IA	All alpha proteins	
2OKHA		
2AN6A	All beta proteins	TRAF domain-like
1GNHA	All beta proteins	Concanavalin A-like lectins/gl

		ucanases
2D7HD		
2BJ1A	All alpha proteins	Ribbon-helix-helix Ferredoxin-like
2IA9C		SpoVG-like
1KQ5A	All beta proteins	Single-stranded right-handed beta-helix
1N32F	Alpha and beta proteins (a+b)	Ferredoxin-like
1N32L	All beta proteins	OB-fold
1B26A	Alpha and beta proteins (a/b)	NAD(P)-binding Rossmann-fold domains Aminoacid dehydrogenase-like N-terminal domain
1S60A	Alpha and beta proteins (a+b)	Acyl-CoA N-acyltransferases (Nat)
1R8YF	Alpha and beta proteins (a/b)	S-adenosyl-L-methionine-dependent methyltransferases
2ERJF	All beta proteins	Immunoglobulin-like beta-sandwich Immunoglobulin-like beta-sandwich
2MEV1	All beta proteins	Nucleoplasmin-like/VP (viral coat and capsid proteins)
2ZAGD		
1CJKA	Alpha and beta proteins (a+b)	Ferredoxin-like
2IF7A		
1T62A	All beta proteins	PUA domain-like
1ZBE1		
2C8TA		
1JN0A	Alpha and beta proteins (a/b)	NAD(P)-binding Rossmann-fold domains NAD(P)-binding Rossmann-fold domains FwdE/GAPDH domain-like
1X3GB		
2FDMA		TIM beta/alpha-barrel TIM beta/alpha-barrel FKBP-like
2BT8A		
2EX3A	Alpha and beta proteins (a/b)	Ribonuclease H-like motif DNA/RNA polymerases
2BNYA		

1WG3A		Immunoglobulin-like beta-sandw ich
1QATA	All alpha proteins	EF Hand-like C2 domain-like TIM beta/alpha-barrel
1TP8A	All beta proteins	
1AUYB	All beta proteins	Nucleoplasmin-like/VP (viral c oat and capsid proteins)
2BR7A		
2JBKA	All alpha proteins	N-cbl like The 'swivelling' beta/beta/alpha domain Phosp horylase/hydrolase-like Phos phorylase/hydrolase-like
1IM0A	Membrane and cell surface	Transmembrane beta-barrels
3BI9X		
2NUAE		Flavodoxin-like ATP-grasp
1XHUB	Alpha and beta proteins (a/b)	Restriction endonuclease-like
1GLEG	Alpha and beta proteins (a/b)	Ribonuclease H-like motif Ri bonuclease H-like motif
1XXDC	All beta proteins	Ecotin trypsin inhibitor
2GLUA	Alpha and beta proteins (a/b)	S-adenosyl-L-methionine-depend ent methyltransferases
2IZWB		
1P4LD	Alpha and beta proteins (a+b)	C-type lectin-like
1XZQB	Alpha and beta proteins (a+b)	Folate-binding domain
2ODJA		
2FGSA		
2GY5A		
2PJRB		
2P6TG		
1FG9C	All beta proteins	Immunoglobulin-like beta-sandw ich Immunoglobulin-like beta -sandwich
1J7VR	All beta proteins	Immunoglobulin-like beta-sandw ich Immunoglobulin-like beta -sandwich
1C9IA	All alpha proteins	alpha-alpha superhelix 7-bla ded beta-propeller

1N8BA	All beta proteins	Baseplate structural protein gp8
1KAWA	All beta proteins	OB-fold
1YC61	All beta proteins	Nucleoplasmin-like/VP (viral coat and capsid proteins)
2Q7OE		Phosphorylase/hydrolase-like
2ISLA		
1TGHA	Alpha and beta proteins (a+b)	TBP-like TBP-like
1JN1A	Alpha and beta proteins (a+b)	Bacillus chorismate mutase-like
1PRTF	All beta proteins	OB-fold
1PRTD	All beta proteins	OB-fold
1T9GB	All alpha proteins	Bromodomain-like Acyl-CoA dehydrogenase NM domain-like
3C5PB		
2O3OL		
2OOYD		AMPKBI-like
2OOYA		TBP-like
2RFKA		PUA domain-like Pseudouridine synthase
1Y56B		
2B5LC		
1VRSB	All beta proteins	Immunoglobulin-like beta-sandwich
1LQLC	Alpha and beta proteins (a+b)	OsmC-like
1TTUA	All beta proteins	Immunoglobulin-like beta-sandwich Common fold of diphtheria toxin/transcription factors/cytochrome f beta-Trefoil
1UEXB	Alpha and beta proteins (a+b)	C-type lectin-like
1TNRA	All beta proteins	TNF-like
1Q6WC	Alpha and beta proteins (a+b)	Thioesterase/thiol ester dehydrase-isomerase
1ZGLB	All beta proteins	Immunoglobulin-like beta-sandwich MHC antigen-recognition domain

1YEWA		
1VGUB	Alpha and beta proteins (a/b)	Nucleotide-diphospho-sugar transferases
2C37A		Ribosomal protein S5 domain 2-like Ribonuclease PH domain 2-like
2D5HA		
2IBOA	Alpha and beta proteins (a+b)	Ferredoxin-like
2RALB		Common fold of diphtheria toxin/transcription factors/cytochrome f Common fold of diphtheria toxin/transcription factors/cytochrome f
1OF5A	Alpha and beta proteins (a+b)	Cystatin-like
1OF5B	Alpha and beta proteins (a+b)	Cystatin-like
2Z4HB		
2AWOD	All beta proteins	OB-fold P-loop containing nucleoside triphosphate hydrolases
2R9BA		Acid proteases
2QKMG		
2OPBA		
2JEUA		
3BDRA		
2VHFB		
1CIDA	All beta proteins	Immunoglobulin-like beta-sandwich Immunoglobulin-like beta-sandwich
1M7UB	All beta proteins	Common fold of diphtheria toxin/transcription factors/cytochrome f
1T6YB	All beta proteins	Reductase/isomerase/elongation factor common domain Adenine nucleotide alpha hydrolase-like
2I5BB		
1K3EB	Alpha and beta proteins (a+b)	Secretion chaperone-like
1I1AC	All beta proteins	Immunoglobulin-like beta-sandwich Immunoglobulin-like beta-sandwich

1SJ5B		
1F2NA	All beta proteins	Nucleoplasmin-like/VP (viral coat and capsid proteins)
1WTHD	All beta proteins	Phage tail proteins Phage tail proteins
2C9KA		
1MPYA	Alpha and beta proteins (a+b)	Glyoxalase/Bleomycin resistance protein/Dihydroxybiphenyl dioxygenase Glyoxalase/Bleomycin resistance protein/Dihydroxybiphenyl dioxygenase
1Z50A		
2I3CA		Phosphorylase/hydrolase-like
1XXJB	Alpha and beta proteins (a+b)	T-fold T-fold
2IUNA		
2F8VT		
1B98M	Small proteins	Cystine-knot cytokines
2R61A		OB-fold beta-Grasp (ubiquitin-like)
2REFA		
2V3MA		
1JC7A	All beta proteins	OB-fold
2CY9A	Alpha and beta proteins (a+b)	Thioesterase/thiol ester dehydrase-isomerase
2Z9HC		OB-fold
2F1VA		
2NWAA		PUA domain-like
2OK7C		
1NSLC	Alpha and beta proteins (a+b)	Acyl-CoA N-acyltransferases (Nat)
1VH3C	Alpha and beta proteins (a/b)	Nucleotide-diphospho-sugar transferases
2BSEA		
2HTHB	All beta proteins	PH domain-like barrel
2CCZB	All beta proteins	OB-fold

1I69B	Alpha and beta proteins (a/b)	Periplasmic binding protein-like II
1TLTA	Alpha and beta proteins (a/b)	NAD(P)-binding Rossmann-fold domains NAD(P)-binding Rossmann-fold domains FwdE/GAPDH domain-like
1X36A		
1KGYA	All beta proteins	Galactose-binding domain-like
1XQ4C	All beta proteins	Immunoglobulin-like beta-sandwich
2Z65C		
2FKDA		
2HZSD		
3B7KB		
1QX6A	All beta proteins	Sortase
1ZHIA	All beta proteins	SH3-like barrel
2AMPA	All beta proteins	Trypsin-like serine proteases
2C35B	All beta proteins	OB-fold Dodecin subunit-like
1CI0A	All beta proteins	Split barrel-like
2BA1A		
2HJ0A		
1RI2A	Alpha and beta proteins (a/b)	S-adenosyl-L-methionine-dependent methyltransferases
2EWJA	Multi-domain proteins	Replication terminator protein (Tus)
1I7FA	Alpha and beta proteins (a+b)	Hsp33 domain
2VP1A		
1HCFX	All beta proteins	Immunoglobulin-like beta-sandwich
2ID5B		
1NCNA	All beta proteins	Immunoglobulin-like beta-sandwich
2I46A		
3C0UB		
1IPKB	All beta proteins	Double-stranded beta-helix Double-stranded beta-helix

1EXCA	Alpha and beta proteins (a/b)	Anticodon-binding domain-like
1XJ5A	Alpha and beta proteins (a/b)	S-adenosyl-L-methionine-depend ent methyltransferases
1KP0A	Alpha and beta proteins (a/b)	Ribonuclease H-like motif Cr eatinase/aminopeptidase
1W3GA		
1EF7A	Alpha and beta proteins (a+b)	Cysteine proteinases
1HKXI	Alpha and beta proteins (a+b)	Cystatin-like
2YWQC		Ribosome binding protein Y (Yf iA homologue)
1ORHA	Alpha and beta proteins (a/b)	S-adenosyl-L-methionine-depend ent methyltransferases
2IWLX		
2R76A		
2QLVA		TBP-like
1I78B	Membrane and cell surface	Transmembrane beta-barrels
3BY7E		
1ML8A	Alpha and beta proteins (a+b)	OsmC-like
3BF2A		
2P4VA		
2J3RA		
1KCGC	Alpha and beta proteins (a+b)	MHC antigen-recognition domain
2QWVA		alpha/beta knot
1FOD2	All beta proteins	
2HY3A		
2CA1A	Alpha and beta proteins (a+b)	Nucleocapsid protein dimerizat ion domain
2A8QA	Alpha and beta proteins (a+b)	Nudix
2R3UC		Trypsin-like serine proteases
1ULJA	All beta proteins	ISP domain TBP-like
1AVGI	All beta proteins	Lipocalins
2DZYA		
1J5QA	All beta proteins	Nucleoplasmin-like/VP (viral c

		oat and capsid proteins) Nuc leoplasmin-like/VP (viral coat and capsid proteins)
2O6IB	All alpha proteins	HD-domain/PDEase-like
2GCJC	All beta proteins	PH domain-like barrel
2FA3A		
2E2XA		
2CJRB		Nucleocapsid protein dimerizat ion domain
2R9AB		
2B25B	Alpha and beta proteins (a/b)	S-adenosyl-L-methionine-depend ent methyltransferases
2AGCA		
2P1JA		
1VRLA	All alpha proteins	DNA-glycosylase Nudix
1UT4A	All beta proteins	NAC domain
1BDFA	Alpha and beta proteins (a+b)	DCoH-like DCoH-like Insert subdomain of RNA polymerase a lpha subunit
2BNGA		Cystatin-like
2FFSA	Alpha and beta proteins (a+b)	TBP-like
2DX0B		
2OL5A		
2R86A		PreATP-grasp domain ATP-gras p
1XBRA	All beta proteins	Common fold of diphtheria toxi n/transcription factors/cytoch rome f
2HJWA		
1GUOC	All beta proteins	OB-fold
2HTIA	All beta proteins	Split barrel-like
2OGGA		Chorismate lyase-like
1JSGA	All beta proteins	Oncogene products
2HDIB		
1RM1B	All alpha proteins	Transcription factor IIA (TFII A) alpha-helical domain Tr

			anscription factor IIA (TFIIA) beta-barrel domain
1D2PA	All beta proteins		
1Z9WA	Alpha and beta proteins (a+b)	T-fold	
2DWRA			
1VHIB	Alpha and beta proteins (a+b)	Ferredoxin-like	
1BCRB			
1F97A	All beta proteins	Immunoglobulin-like beta-sandwich Immunoglobulin-like beta-sandwich	
1EJFA	All beta proteins	HSP20-like chaperones	
3CNWA		TBP-like	
2QEAA			
1U8SB	Alpha and beta proteins (a+b)	Ferredoxin-like Ferredoxin-like	
1CY0A	Multi-domain proteins	Prokaryotic type I DNA topoisomerase	
1G5XA	Alpha and beta proteins (a/b)	Thiolase-like Thiolase-like	
1GZ5A	Alpha and beta proteins (a/b)	UDP-Glycosyltransferase/glycogen phosphorylase	
2EG0A			
3BPSP			
2BSBA			
2BHMA		Cystatin-like	
1T94A	Alpha and beta proteins (a+b)	Lesion bypass DNA polymerase (Y-family) little finger domain DNA/RNA polymerases	
2PCSA		TBP-like	
2PT7G			
2D13B	Alpha and beta proteins (a/b)	Adenine nucleotide alpha hydrolase-like	
1XK5A			
1XA7A	Multi-domain proteins	beta-lactamase/transpeptidase-like	
1F6OA	All beta proteins	FMT C-terminal domain-like	
2CFXA	All alpha proteins	DNA/RNA-binding 3-helical bund	

		le Ferredoxin-like
3BNWB		
2CO1A	All beta proteins	Common fold of diphtheria toxin/transcription factors/cytochrome f
2FDOA	Alpha and beta proteins (a+b)	AF2331-like
1VYQA	All beta proteins	beta-clip
2FHDA		
2BE3B	Alpha and beta proteins (a+b)	Nucleotidyltransferase
2GSHA		
2NNFB		
2GJVA	Alpha and beta proteins (a+b)	Phage tail protein-like
2PTUA		
1ZOQA		
1QYND	Alpha and beta proteins (a+b)	SecB-like
3B6YB		
2QPVA		TBP-like
2AUAA	Alpha and beta proteins (a+b)	ADP-ribosylation
1WAMA	Alpha and beta proteins (a/b)	Nucleotide-binding domain Nucleotide-binding domain FAD-linked reductases C-terminal domain
2HJ9C		Profilin-like
2IECB	Alpha and beta proteins (a+b)	MK0786-like
1OQNB	All beta proteins	PH domain-like barrel
2EGIG		
1UOCB	Alpha and beta proteins (a/b)	Ribonuclease H-like motif
1CR5A	All beta proteins	Double psi beta-barrel Cdc48 domain 2-like
1S7JA	Alpha and beta proteins (a+b)	Diaminopimelate epimerase-like
1EDYB	All beta proteins	Common fold of diphtheria toxin/transcription factors/cytochrome f
1YOXB	All beta proteins	Double-stranded beta-helix
1Q67B	All beta proteins	PH domain-like barrel

2JEVA	Alpha and beta proteins (a+b)	Acyl-CoA N-acyltransferases (Nat)
2F1XA		
1NB8A	Alpha and beta proteins (a+b)	Cysteine proteinases
1HMWA	All alpha proteins	alpha/alpha toroid Hyaluronate lyase-like C-terminal domain Supersandwich
2AEGA	Alpha and beta proteins (a+b)	BB1717-like
2CFAA		
2IN5B		YmcC-like
2J2ZB		Common fold of diphtheria toxin/transcription factors/cytochrome f
2J3LB		
1GTQA	Alpha and beta proteins (a+b)	T-fold
1KR2F	Alpha and beta proteins (a/b)	Adenine nucleotide alpha hydrolase-like
2CM9A		
2I1SA		MM3350-like
1SMPI	All beta proteins	Streptavidin-like
2O7VA		
2CH8A		
2HGVA		
2PW6A		Phosphorylase/hydrolase-like
2V72A		
2ACAA	Alpha and beta proteins (a+b)	CYTH-like phosphatases
1H6XA	All beta proteins	Galactose-binding domain-like
1ZCWA	Alpha and beta proteins (a+b)	Antiparallel beta/alpha barrel (PT-barrel)
2EH1B		
2J58A		
2IQ1A		
2RETD		Pili subunits
2E7DB		

1JSSA	Alpha and beta proteins (a+b)	TBP-like
1EYSH	All beta proteins	PRC-barrel domain Single transmembrane helix
2F5KB	All beta proteins	SH3-like barrel
2YXYA		
2YX1B		
1RZ0A	All beta proteins	Split barrel-like
1PSUB	Alpha and beta proteins (a+b)	Thioesterase/thiol ester dehydrase-isomerase
1P4UA	All beta proteins	Immunoglobulin-like beta-sandwich
1GQPA	All beta proteins	Galactose-binding domain-like
1NMPA	Alpha and beta proteins (a/b)	NIF3 (NGG1p interacting factor 3)-like
1YLNA	All beta proteins	Split barrel-like Split barrel-like
2EXJA	All beta proteins	Concanavalin A-like lectins/glucanases 5-bladed beta-propeller
1EM2A	Alpha and beta proteins (a+b)	TBP-like
1IM3D	All beta proteins	Immunoglobulin-like beta-sandwich
2QZ9A		
2PQFA		
2RJZB		
3CI0K		SAM domain-like SAM domain-like Pili subunits Pili subunits
3CI0I		Pili subunits
1G43A	All beta proteins	Common fold of diphtheria toxin/transcription factors/cytochrome f
1IM8B	Alpha and beta proteins (a/b)	S-adenosyl-L-methionine-dependent methyltransferases
1PNGA	All beta proteins	Nucleoplasmin-like/VP (viral coat and capsid proteins) Nucleoplasmin-like/VP (viral coat and capsid proteins)

1EPBA	All beta proteins	Lipocalins
1WQLB	Alpha and beta proteins (a+b)	Cystatin-like
1KWIA	Alpha and beta proteins (a+b)	Cystatin-like
2ZF4E		
2F7CA		
1ZVNA		
1VH9A	Alpha and beta proteins (a+b)	Thioesterase/thiol ester dehyd rase-isomerase
1SX8A	Alpha and beta proteins (a/b)	Restriction endonuclease-like
1USUB	Alpha and beta proteins (a+b)	Aha1/BPI domain-like
2QWZD		
1K3BA	All beta proteins	Streptavidin-like
2V1LA		
2B59A	All beta proteins	Common fold of diphtheria toxi n/transcription factors/cytoch rome f
1PQZA	All beta proteins	Immunoglobulin-like beta-sandw ich MHC antigen-recognition domain
3BM2B		
1UN1A	All beta proteins	Concanavalin A-like lectins/gl ucanases
2ZMFA		
1VJHA	Alpha and beta proteins (a+b)	TBP-like
3BCZA		
2P19A		Chorismate lyase-like
1OTGA	Alpha and beta proteins (a+b)	Tautomerase/MIF
1T4WA	All beta proteins	Common fold of diphtheria toxi n/transcription factors/cytoch rome f
2OV2J		
1XMXA	Alpha and beta proteins (a/b)	Restriction endonuclease-like
1VR4A	Alpha and beta proteins (a+b)	Dodecin subunit-like
2D42A		
1Y7MA	All beta proteins	L D-transpeptidase catalytic

		domain-like LysM domain
2FXVA	Alpha and beta proteins (a/b)	PRTase-like
3BTNA		
2P0LA		Class II aaRS and biotin synth etases
2QGOA		
2RK0A		
2QYZA		
2R39A		
2DR3A		
3B47A		
1TE5A	Alpha and beta proteins (a+b)	Ntn hydrolase-like
1Q2YA	Alpha and beta proteins (a+b)	Acyl-CoA N-acyltransferases (N at)
1ZNOB	Alpha and beta proteins (a/b)	Anticodon-binding domain-like
1ROWA	All beta proteins	Immunoglobulin-like beta-sandw ich
2HP7A		
3BRNB		
1ALYA	All beta proteins	TNF-like
2Z0BB		
2CZVD		Ferredoxin-like
1TY2B	All beta proteins	OB-fold beta-Grasp (ubiquiti n-like)
2A8IA		
1DUEA	All beta proteins	Trypsin-like serine proteases
1D3BF	All beta proteins	Sm-like fold
1DQTA	All beta proteins	Immunoglobulin-like beta-sandw ich
1MI8A	All beta proteins	Hedgehog/intein (Hint) domain
1DFAA	All beta proteins	
2HQLA		
1T5RC	Membrane and cell surface	Leukocidin-like

1RFEA	All beta proteins	Split barrel-like
2ERVA		
2RDXE		
2HQ7B	All beta proteins	Split barrel-like
1FVIA	All beta proteins	OB-fold ATP-grasp
2F4WB	Alpha and beta proteins (a+b)	UBC-like
1MKBA	Alpha and beta proteins (a+b)	Thioesterase/thiol ester dehydrase-isomerase
3C26A		
1VJNA	Alpha and beta proteins (a+b)	Metallo-hydrolase/oxidoreductase
1ESOA	All beta proteins	Immunoglobulin-like beta-sandwich
1YZVA		
1UZ0A	All beta proteins	Galactose-binding domain-like
2AMUA		
1WMYA	Alpha and beta proteins (a+b)	C-type lectin-like
2E7YA		Metallo-hydrolase/oxidoreductase
1LG7A	Alpha and beta proteins (a+b)	VSV matrix protein
2PKHB		Chorismate lyase-like
1Y71B	All beta proteins	SH3-like barrel
2NZCD		Ferredoxin-like
1ZDEA		
1LJOA	All beta proteins	Sm-like fold
1Y12C	All beta proteins	Hcp1-like
1VCHE	Alpha and beta proteins (a/b)	PRTase-like
2QC1B		
2PFRB		
2E7VA		
2QHKA		
2FD6U		Snake toxin-like Snake toxin-like Snake toxin-like

1XBWA	Alpha and beta proteins (a+b)	Ferredoxin-like
2FBLA	Alpha and beta proteins (a+b)	CYTH-like phosphatases
1Z9OA		
1AT0A	All beta proteins	Hedgehog/intein (Hint) domain
1NEUA	All beta proteins	Immunoglobulin-like beta-sandwich
2OVSA		
1QA7A	All beta proteins	Trypsin-like serine proteases
1QOUB	All beta proteins	PEBP-like
1STMA	All beta proteins	Nucleoplasmin-like/VP (viral coat and capsid proteins)
1QCSA	All beta proteins	Double psi beta-barrel Cdc48 domain 2-like
1JHSA	Alpha and beta proteins (a+b)	Mog1p/PsbP-like
2QCKA		
1NH2C	All beta proteins	Transcription factor IIA (TFIIA) beta-barrel domain
2VGAA		
2GWLA		
2AZWA	Alpha and beta proteins (a+b)	Nudix
1LSHB	Membrane and cell surface	Lipovitellin-phosvitin complex; beta-sheet shell regions
2IHYB		
1T9MA	All beta proteins	Split barrel-like
2J6RA		
2P0AA		
1ZLEB		
1UNND	Alpha and beta proteins (a+b)	Lesion bypass DNA polymerase (Y-family) little finger domain
1USPA	Alpha and beta proteins (a+b)	OsmC-like
1SFPA	All beta proteins	CUB-like
1H9RA	All beta proteins	OB-fold OB-fold
1VKBA	Alpha and beta proteins (a+b)	Gamma-glutamyl cyclotransferase-like

1R75A	All beta proteins	Smp-1-like
1UZXA	Alpha and beta proteins (a+b)	UBC-like
1PK6A	All beta proteins	TNF-like
2ASUB		
1O6AB	All beta proteins	Surface presentation of antigens (SPOA)
2P7HB		S-adenosyl-L-methionine-dependent methyltransferases
1TZ0A	Alpha and beta proteins (a+b)	Ferredoxin-like
2Z13A		
2PWWA		TBP-like
2HNFA		
2HFNJ		
1VHSB	Alpha and beta proteins (a+b)	Acyl-CoA N-acyltransferases (Nat)
1LUZA	All beta proteins	OB-fold
1N9PA	All beta proteins	Immunoglobulin-like beta-sandwich
2QH9B		
1S7IA	Alpha and beta proteins (a+b)	Ferredoxin-like
2J22A		
2IIAA		
1ZN6A	Alpha and beta proteins (a+b)	BB1717-like
2EKYA		
2OQ8A		
1KVDB	Alpha and beta proteins (a+b)	
2C2IA	Alpha and beta proteins (a+b)	Thioesterase/thiol ester dehydrase-isomerase
2HQYA		Acyl-CoA N-acyltransferases (Nat) Acyl-CoA N-acyltransferases (Nat)
1VM0A	Alpha and beta proteins (a+b)	IF3-like
2I02A	All beta proteins	Split barrel-like
2R2CB		

2ACOA		
1YX1B	Alpha and beta proteins (a/b)	TIM beta/alpha-barrel
2Q03B		AOC barrel-like
1GBGA	All beta proteins	Concanavalin A-like lectins/glucanases
2RGQB		Cystatin-like
1ZVCA	All beta proteins	AOC barrel-like
1RGXC	Small proteins	Resistin
2OD5A		DNA/RNA-binding 3-helical bundle
2OYAA		
2NYUB		
1VI3A	All beta proteins	PEBP-like
1R77B		
2EX4A	Alpha and beta proteins (a/b)	S-adenosyl-L-methionine-dependent methyltransferases
1R0UA	All beta proteins	Lipocalins
2FK9A		
1MDCA	All beta proteins	Lipocalins
2FE3B		
3CLAA	Alpha and beta proteins (a/b)	CoA-dependent acyltransferases
3BLZF		Cystatin-like
1VR8A	Alpha and beta proteins (a+b)	TM1622-like
1PCFA	Alpha and beta proteins (a+b)	ssDNA-binding transcriptional regulator domain
3CK1A		
1DDWA	All beta proteins	PH domain-like barrel
1QSTA	Alpha and beta proteins (a+b)	Acyl-CoA N-acyltransferases (Nat)
1JUVA	Alpha and beta proteins (a/b)	Dihydrofolate reductase-like
1F3UH	All beta proteins	triple barrel
2E12A		
2FCKA	Alpha and beta proteins (a+b)	Acyl-CoA N-acyltransferases (N

		at)
1OA8D	All beta proteins	AXH domain
2PY2A		
2O99A	Alpha and beta proteins (a+b)	Profilin-like
2PLWA		
2JGBA		
2BOPA	Alpha and beta proteins (a+b)	Ferredoxin-like
2VLGD		
1S5UG	Alpha and beta proteins (a+b)	Thioesterase/thiol ester dehyd rase-isomerase
1FLTX	All beta proteins	Immunoglobulin-like beta-sandw ich
1NEPA	All beta proteins	Immunoglobulin-like beta-sandw ich
2DTCB		
3B7CA		Cystatin-like
1PFVA	All alpha proteins	
1VMBA	Alpha and beta proteins (a+b)	Ferredoxin-like
1U5DA	All beta proteins	PH domain-like barrel
2OQBB		
2QIYB		Cystatin-like
1TH7A	All beta proteins	Sm-like fold
2A15A	Alpha and beta proteins (a+b)	Cystatin-like
1UU5A	All beta proteins	Concanavalin A-like lectins/gl ucanases
1T0PB		
2O30B		
2ISBA	Alpha and beta proteins (a/b)	The 'swivelling' beta/beta/alp ha domain
2OITA		
2OKMA		
1IBYA	All beta proteins	Cupredoxin-like
2AJ6A	Alpha and beta proteins (a+b)	Acyl-CoA N-acyltransferases (N at)

1VJFA	Alpha and beta proteins (a+b)	YbaK/ProRS associated domain
1IUJB	Alpha and beta proteins (a+b)	Ferredoxin-like
1M4JA	Alpha and beta proteins (a+b)	Gelsolin-like
3CMBB		Acetoacetate decarboxylase-lik e
1KAFE	Alpha and beta proteins (a+b)	MotA C-terminal domain-like
1TVGA	All beta proteins	Galactose-binding domain-like
2HBTA		
1O26A	Alpha and beta proteins (a+b)	Thymidylate synthase-complemen ting protein Thy1
1GY7C	Alpha and beta proteins (a+b)	Cystatin-like
2D3MA		
2CIUA		
2Z6OA		
1T9IB	Alpha and beta proteins (a+b)	Homing endonuclease-like
1V2BB	Alpha and beta proteins (a+b)	Mog1p/PsbP-like
2R78A		
2ASFA	All beta proteins	Split barrel-like
2R4IA		Cystatin-like
2A7MA		
3BCWA		
2ITEA		Immunoglobulin-like beta-sandw ich
2DT4A		
2C6UA		
2HTDB		
2BO9B	Alpha and beta proteins (a+b)	Cystatin-like Cystatin-like
2JDCA		Acyl-CoA N-acyltransferases (N at)
2CDPD		
2IA1B		BH3703-like
1W0HA	Alpha and beta proteins (a/b)	Ribonuclease H-like motif

1XS0A	Alpha and beta proteins (a+b)	Inhibitor of vertebrate lysozyme Ivy
2DTJA		
1JOVA	All beta proteins	Supersandwich
2ASKA		
1W4SA		
2Q2IB		
3BA3A		
2BEMA	All beta proteins	Immunoglobulin-like beta-sandwich
1VHWA	Alpha and beta proteins (a/b)	Phosphorylase/hydrolase-like
3BP1A		
1ZHVA	Alpha and beta proteins (a+b)	Ferredoxin-like Ferredoxin-like
2RA6A		
2V6VA		
1FL0A	All beta proteins	OB-fold
1JL0B	Alpha and beta proteins (a+b)	S-adenosylmethionine decarboxylase
1D7PM	All beta proteins	Galactose-binding domain-like
2PRXB		
1LMIA	All beta proteins	Immunoglobulin-like beta-sandwich
2O6PA		Immunoglobulin-like beta-sandwich
1DP7P	All alpha proteins	DNA/RNA-binding 3-helical bundle
1DFMB	Alpha and beta proteins (a/b)	Restriction endonuclease-like
2OA9B		
1TP6A	Alpha and beta proteins (a+b)	Cystatin-like
1F7LA	Alpha and beta proteins (a+b)	4'-phosphopantetheinyl transferase
1T61C	Alpha and beta proteins (a+b)	C-type lectin-like C-type lectin-like
1UV4A	All beta proteins	5-bladed beta-propeller

2IMJA		Cystatin-like
1LO7A	Alpha and beta proteins (a+b)	Thioesterase/thiol ester dehydrase-isomerase
1QWOA	Alpha and beta proteins (a/b)	Phosphoglycerate mutase-like
1NF9A	Alpha and beta proteins (a/b)	Isochorismatase-like hydrolases
2OPLB		OsmC-like
2QSWA		Ferredoxin-like
2J12A	All beta proteins	Virus attachment protein globular domain
2QL8A		
1Z1SA		Cystatin-like
1DZKA	All beta proteins	Lipocalins
1WPUA	Alpha and beta proteins (a+b)	Hut operon positive regulatory protein HutP
3SEBA	All beta proteins	OB-fold beta-Grasp (ubiquitin-like)
1UPQA	All beta proteins	PH domain-like barrel
1GV9A	All beta proteins	Concanavalin A-like lectins/glucanases
2RL8A		Mannose 6-phosphate receptor domain
2PQ8A		
1IDPA	Alpha and beta proteins (a+b)	Cystatin-like
2QFEA		
2RB8A		
2HEWF	All beta proteins	TNF-like
2F5TX		Sm-like fold Phospholipase D/nuclease
2NN5A		TBP-like
2J8MB		
2CJTA	All beta proteins	C2 domain-like
2DPLB		
1PP0B	Alpha and beta proteins (a+b)	CytB endotoxin-like
2PQXA		

1PKHB	All beta proteins	beta-clip
1NOFA	All beta proteins	Glycosyl hydrolase domain Gl ycosyl hydrolase domain TIM beta/alpha-barrel
2BWQA	All beta proteins	C2 domain-like
1V30A	Alpha and beta proteins (a+b)	Gamma-glutamyl cyclotransferas e-like
1ES5A	Multi-domain proteins	beta-lactamase/transpeptidase-like
1G8AA	Alpha and beta proteins (a/b)	S-adenosyl-L-methionine-depend ent methyltransferases
1NYCA	All beta proteins	Streptavidin-like
2EW0A	Alpha and beta proteins (a+b)	VC0467-like
1PFBA	All beta proteins	SH3-like barrel
1F8EA	All beta proteins	6-bladed beta-propeller
1N13B	Alpha and beta proteins (a+b)	
1UWWB	All beta proteins	Galactose-binding domain-like
2B06A	Alpha and beta proteins (a+b)	Nudix
2OCTB		
2BU3A	Alpha and beta proteins (a+b)	Cysteine proteinases
2V0UA		
1Q5YD	Alpha and beta proteins (a+b)	Ferredoxin-like
2J73A		Prealbumin-like
1VJUB	Alpha and beta proteins (a+b)	Coproporphyrinogen III oxidase
1LLFA	Alpha and beta proteins (a/b)	alpha/beta-Hydrolases
1H2WA	All beta proteins	7-bladed beta-propeller alph a/beta-Hydrolases
2NUHA		
1K55C	Multi-domain proteins	beta-lactamase/transpeptidase-like
3B79A		
1WCKA		
2P8GA		
2HD9A		PUA domain-like

1PB7A	Alpha and beta proteins (a/b)	Periplasmic binding protein-like II
1KUFA	Alpha and beta proteins (a+b)	Zincin-like
2I5FA		PH domain-like barrel
2EHZA		
2NN8A	All beta proteins	Concanavalin A-like lectins/glucanases
2QIMA		
1SJWA	Alpha and beta proteins (a+b)	Cystatin-like
2JDAB		
1OH4A	All beta proteins	Galactose-binding domain-like
1EAJB	All beta proteins	Immunoglobulin-like beta-sandwich
2QIKA		
3BFQG		
1GPIA	All beta proteins	Concanavalin A-like lectins/glucanases
1MKKA	Small proteins	Cystine-knot cytokines
1V9YA	Alpha and beta proteins (a+b)	Profilin-like
2Q4NA	All beta proteins	Lipocalins
2VC8A		
2RDQA		
3BLNA		
2PV1A		FKBP-like
1JL1A	Alpha and beta proteins (a/b)	Ribonuclease H-like motif
1F9VA	Alpha and beta proteins (a/b)	P-loop containing nucleoside triphosphate hydrolases
2IC2B	All beta proteins	Immunoglobulin-like beta-sandwich
1UCDA	Alpha and beta proteins (a+b)	Ribonuclease Rh-like
1ZCEA	All beta proteins	PUA domain-like
2AGKA		
2D5WA		

1YG9A		
2D1SA		
1HYOA	All beta proteins	SH3-like barrel FAH
2IMQX		DNase I-like
2NLVB	Alpha and beta proteins (a+b)	XisI-like
2R5OA		
2GZQA		
2G8MA		
1FLMA	All beta proteins	Split barrel-like
2F69A	All beta proteins	open-sided beta-meander beta -clip
1WVFA	Alpha and beta proteins (a+b)	Ferredoxin-like FAD-binding/ transporter-associated domain-like
2J9CA		
2CCVA	All beta proteins	HPA-like
1LQ9A	Alpha and beta proteins (a+b)	Ferredoxin-like
1DG6A	All beta proteins	TNF-like
2OKTA		
1QDDA	Alpha and beta proteins (a+b)	C-type lectin-like
1PQHB	All beta proteins	Double psi beta-barrel
2DXUA		
2CC6A	Alpha and beta proteins (a+b)	Dodecin subunit-like
1GXUA	Alpha and beta proteins (a+b)	Ferredoxin-like
2DY0A		
2G3RA	All beta proteins	SH3-like barrel SH3-like bar rel
2FVVA	Alpha and beta proteins (a+b)	Nudix
2COVF		
2JCQA		
2B3NB	Alpha and beta proteins (a+b)	
2VBKA		
1USCA	All beta proteins	Split barrel-like

1W7CA		Supersandwich Cystatin-like Cystatin-like
3C8LA		
1KYFA	All beta proteins	Immunoglobulin-like beta-sandwich Subdomain of clathrin and coatomer appendage domain
1MJUL	All beta proteins	Immunoglobulin-like beta-sandwich Immunoglobulin-like beta-sandwich
3B5MA		
1LF7A	All beta proteins	Lipocalins
1O7IB	All beta proteins	OB-fold
1WN2A		
3BUUA		
2FLHD		
2ZEXA		
2BMOB	Alpha and beta proteins (a+b)	Cystatin-like
2DLBA		YopT-like
1NWWB	Alpha and beta proteins (a+b)	Cystatin-like
1WC2A		Double psi beta-barrel
2QF4A		
1Z0WA		
1J98A	Alpha and beta proteins (a+b)	LuxS/MPP-like metallohydrolase
1M15A	All alpha proteins	Guanido kinase N-terminal domain Glutamine synthetase/guanido kinase
1VR7A	Alpha and beta proteins (a+b)	S-adenosylmethionine decarboxylase
2FRGP		
2OW6A		immunoglobulin/albumin-binding domain-like Supersandwich 7-stranded beta/alpha barrel
1KQ6A	Alpha and beta proteins (a+b)	PX domain
2G7BA		
2RFRA		Cystatin-like

1H4XA	Alpha and beta proteins (a/b)	SpoIIaa-like
2FHZB	Alpha and beta proteins (a+b)	Colicin D/E5 nuclease domain
2AWKA		
2BWFA	Alpha and beta proteins (a+b)	beta-Grasp (ubiquitin-like)
1E9GA	All beta proteins	OB-fold
2O9UX	Alpha and beta proteins (a+b)	Cystatin-like
1GWMA	All beta proteins	Galactose-binding domain-like
1XT5A		
2BZVA		
2OLNA		
1T3YA	Alpha and beta proteins (a+b)	Gelsolin-like
1U07B	Alpha and beta proteins (a+b)	TolA/TonB C-terminal domain
2EABA		
1KT6A	All beta proteins	Lipocalins
1OH0A	Alpha and beta proteins (a+b)	Cystatin-like
1H4GB	All beta proteins	Concanavalin A-like lectins/glucanases
1YS1X		
1T2DA	Alpha and beta proteins (a/b)	NAD(P)-binding Rossmann-fold domains LDH C-terminal domain-like
1F86A	All beta proteins	Prealbumin-like
1Z2UA	Alpha and beta proteins (a+b)	UBC-like
1N62C	Alpha and beta proteins (a+b)	CO dehydrogenase flavoprotein C-domain-like FAD-binding/transporter-associated domain-like
1K5NA	All beta proteins	Immunoglobulin-like beta-sandwich MHC antigen-recognition domain
1K5NB	All beta proteins	Immunoglobulin-like beta-sandwich
2CARB	Alpha and beta proteins (a/b)	Anticodon-binding domain-like
1QLWA	Alpha and beta proteins (a/b)	alpha/beta-Hydrolases
2H5OA		

1W66A	Alpha and beta proteins (a+b)	Class II aaRS and biotin synthetases
2FFYA	Multi-domain proteins	beta-lactamase/transpeptidase-like
2O90A		
1PMHX	All beta proteins	Galactose-binding domain-like
2AXWA		Common fold of diphtheria toxin/transcription factors/cytochrome f
1EUWA	All beta proteins	beta-clip
2D5MA		
1LQTA	Alpha and beta proteins (a/b)	FAD/NAD(P)-binding domain Nucleotide-binding domain Nucleotide-binding domain
1KMVA	Alpha and beta proteins (a/b)	Dihydrofolate reductase-like
1RQWA	All beta proteins	Osmotin thaumatin-like protein
2AU7A		
1FSGA	Alpha and beta proteins (a/b)	PRTase-like
2OIZD		
1D5TA	Alpha and beta proteins (a/b)	FAD/NAD(P)-binding domain FAD/NAD(P)-binding domain FAD-linked reductases C-terminal domain
1UOWA	All beta proteins	C2 domain-like
2GJ3B		
2R16A		
1Y55X		
2QCPX		
2H3LB	All beta proteins	PDZ domain-like
1NQJB	All beta proteins	CUB-like
1LKKA	Alpha and beta proteins (a+b)	SH2-like
1OD3A	All beta proteins	Galactose-binding domain-like
2A6ZA	All beta proteins	Concanavalin A-like lectins/glucanases
2JHFA	All beta proteins	GroES-like GroES-like NAD(P)-binding Rossmann-fold domai

		ns
1TT8A	Alpha and beta proteins (a+b)	Chorismate lyase-like
2PNDA		
2GGCA	Alpha and beta proteins (a+b)	Creatinase/aminopeptidase
2CNQA	Alpha and beta proteins (a+b)	SAICAR synthase-like
1GA6A	Alpha and beta proteins (a/b)	Subtilisin-like
2CWSA		
1O7JA	Alpha and beta proteins (a/b)	Glutaminase/Asparaginase
2CHHA	All beta proteins	Calcium-mediated lectin
1K4IA	Alpha and beta proteins (a+b)	YrdC/RibB
1UNQA	All beta proteins	PH domain-like barrel
1IXHA	Alpha and beta proteins (a/b)	Periplasmic binding protein-li ke II
2VK5A		
1U2HA		
2E4TA		
2Z6WA		Cyclophilin-like
1K5CA	All beta proteins	Single-stranded right-handed b eta-helix
1NKIA	Alpha and beta proteins (a+b)	Glyoxalase/Bleomycin resistanc e protein/Dihydroxybiphenyl di oxygenase
1RTQA	Alpha and beta proteins (a/b)	Phosphorylase/hydrolase-like
1LUGA	All beta proteins	Carbonic anhydrase
2UU8A		Concanavalin A-like lectins/gl ucanases
2BT9A		
1N4WA	Alpha and beta proteins (a/b)	FAD/NAD(P)-binding domain FA D/NAD(P)-binding domain FAD- linked reductases C-terminal domain
1OEWA	All beta proteins	Acid proteases
2BW4A	All beta proteins	Cupredoxin-like Cupredoxin-l ike
1F9YA	Alpha and beta proteins (a+b)	

1GWEA	Multi-domain proteins	Heme-dependent catalase-like
2F01B	All beta proteins	Streptavidin-like
2FMAA		
1PJXA	All beta proteins	6-bladed beta-propeller
1X8QA	All beta proteins	Lipocalins
2HS1A		
2H5CA	All beta proteins	Trypsin-like serine proteases
1W0NA	All beta proteins	Galactose-binding domain-like
1PQ7A	All beta proteins	Trypsin-like serine proteases
